# Machine Learning Models for Predicting Bioavailability of Traditional and Emerging Aromatic Contaminants in Plant Roots

**DOI:** 10.3390/toxics12100737

**Published:** 2024-10-12

**Authors:** Siyuan Li, Yuting Shen, Meng Gao, Huatai Song, Zhanpeng Ge, Qiuyue Zhang, Jiaping Xu, Yu Wang, Hongwen Sun

**Affiliations:** MOE Key Laboratory of Pollution Processes and Environmental Criteria, College of Environmental Science and Engineering, Nankai University, Tianjin 300350, China; lisiyuan@mail.nankai.edu.cn (S.L.); shen-yt24@mails.tsinghua.edu.cn (Y.S.); gaomengnk@mail.nankai.edu.cn (M.G.); 15305440229@163.com (H.S.); g1261841715@163.com (Z.G.); zhangqiuyue@mail.nankai.edu.cn (Q.Z.); xujiapingnk@163.com (J.X.)

**Keywords:** aromatic contaminants, root uptake, root concentration factor, RCF, GBRT, molecular descriptors

## Abstract

To predict the behavior of aromatic contaminants (ACs) in complex soil–plant systems, this study developed machine learning (ML) models to estimate the root concentration factor (RCF) of both traditional (e.g., polycyclic aromatic hydrocarbons, polychlorinated biphenyls) and emerging ACs (e.g., phthalate acid esters, aryl organophosphate esters). Four ML algorithms were employed, trained on a unified RCF dataset comprising 878 data points, covering 6 features of soil–plant cultivation systems and 98 molecular descriptors of 55 chemicals, including 29 emerging ACs. The gradient-boosted regression tree (GBRT) model demonstrated strong predictive performance, with a coefficient of determination (R^2^) of 0.75, a mean absolute error (MAE) of 0.11, and a root mean square error (RMSE) of 0.22, as validated by five-fold cross-validation. Multiple explanatory analyses highlighted the significance of soil organic matter (SOM), plant protein and lipid content, exposure time, and molecular descriptors related to electronegativity distribution pattern (GATS8e) and double-ring structure (fr_bicyclic). An increase in SOM was found to decrease the overall RCF, while other variables showed strong correlations within specific ranges. This GBRT model provides an important tool for assessing the environmental behaviors of ACs in soil–plant systems, thereby supporting further investigations into their ecological and human exposure risks.

## 1. Introduction

Aromatic contaminants (ACs), defined by the presence of one or more aromatic rings in their structure, are important pollutants in the environment [1]. Traditional ACs, such as polycyclic aromatic hydrocarbons (PAHs), polychlorinated biphenyls (PCBs), and organochlorine pesticides, are typically hydrophobic and chemically stable, contributing to their resistance to natural degradation processes [2]. These compounds are widely prevalent in the environment due to human activities, including residential heating [3], incineration [4,5], and industrial processes [6]. These traditional ACs, whether released as primary substances, intermediates, or by-products, are of significant concerns due to their persistence and potential for bioaccumulation [7,8,9]. For example, PAHs, with their fused aromatic ring structures, exhibit low water solubility and a strong affinity for organic matter, leading to their persistence in soils and sediments [10]. Similarly, the biphenyl structure of PCBs, combined with varying degrees of chlorination, increases their lipophilicity and environmental stability, promoting bioaccumulation in the fatty tissues of organisms and resistance to biodegradation [6]. Organochlorine pesticides, such as dichlorodiphenyltrichloroethane, share these characteristics due to their high chlorine content, enabling them to persist in the environment for decades and undergo long-range transport [11].

Recently, several emerging contaminants with aromatic structures, including plasticizers and flame retardants, have been identified in soil–plant systems [12,13]. Unlike traditional ACs, emerging ACs such as poly brominated diphenyl ethers (PBDEs), phthalate acid esters (PAEs), and aryl organophosphate esters (OPEs) often exhibit polar and degradable structural features. PBDEs are flame retardants commonly used in consumer products. These hydrophobic, halogenated compounds have long half-lives, resist degradation, and are prone to bioaccumulation and biomagnification [14]. Despite their strong affinity for sediments, they can travel long distances and persist in both aquatic systems and soils [15]. PAEs, widely used as plasticizers, are easily released into the environment because they are not chemically bound to the plastic matrix [16]. These compounds are commonly found in water, sediment, and soil, and their semi-volatile nature allows them to move through various environmental media [17,18]. Depending on factors such as molecular structure and environmental conditions, the half-lives of PAEs in soil range from less than a week to several months [19]. Aryl OPEs, used as substitutes for organohalogen flame retardants, have been widely detected across various environmental media. Research indicates that emissions of aryl OPEs are increasing [20]. The environmental and health risks posed by these ACs are substantial. PAHs and PCBs are linked to carcinogenic, mutagenic, and endocrine-disrupting effects [21,22]. Similarly, organochlorine pesticides are highly lipophilic, accumulating in the fatty tissues of animals and leading to chronic exposure risks, reproductive harm, and neurological damage [23]. PBDEs are associated with neurodevelopmental toxicity and endocrine disruption [24], while PAEs are linked to reproductive toxicity and developmental disorders in humans and wildlife [25]. Aryl OPEs have been connected to reproductive and neurological dysfunction, as well as genotoxicity [26,27]. Additionally, due to their lipophilic properties, ACs are prone to accumulate in soil and edible plants. The substantial structural differences among various ACs lead to distinct accumulation behaviors in plant roots [28,29,30,31,32,33,34], complicating their migration behaviors in soil–plant systems and making risk assessment more challenging.

The root concentration factor (RCF) is a commonly used metric for evaluating a plant’s ability to accumulate organic compounds, defined as the ratio of the concentration in plant roots to the concentration in soil at equilibrium [35]. Previous studies have shown that the RCF is influenced by chemical properties such as hydrophobicity and sorption–desorption features in the soil–water interface [36,37]. Several factors, such as SOM content, plant species, exposure time, root protein content, root lipid content, cultivation mode, temperature, and transpiration capacity, can influence the RCF of ACs [31,38,39]. Among these, SOM plays a critical role in determining contaminant bioavailability. High SOM levels typically reduce the bioavailability of hydrophobic organic compounds by adsorbing them onto soil particles, thus limiting their mobility and reducing plant uptake. This interaction between SOM and organic contaminants underscores the importance of soil properties in controlling accumulation processes in plant systems [40]. Different plant species exhibit varying capacities to accumulate contaminants, which can be attributed to differences in root structure, protein content, and lipid content. For instance, studies have shown that variations in the PAE absorption among plant species may be linked to differences in their lipid content [41]. Another study found that OPEs can associate with non-specific lipid transfer proteins (nsLTPs), facilitating their absorption by plant roots [42]. While plant exposure time can influence chemical absorption, this effect is often limited under conditions of uniform soil and stable chemical concentrations [37]. Cultivation methods also impact the absorption of contaminants by plant roots [43]. However, many studies have focused primarily on single-factor laboratory investigations, neglecting the combined effects of multiple factors on RCF. While empirical regression models have been widely used to predict RCFs [44], these models traditionally rely on a limited set of physicochemical properties, such as the octanol–water partition coefficient (*K*_ow_) [45]. Compared to similarly halogenated PCBs, PBDEs tend to accumulate more in plant roots due to their higher hydrophobicity, as compounds with high *K*_ow_ values are generally more lipophilic and readily absorbed by roots [46]. Moreover, the relationships between the chemical structures of emerging ACs and their accumulation in plants have not been thoroughly explored. Therefore, the complexity of AC structures and the diverse properties of soil–plant properties necessitate timely and effective approaches to comprehensively assess and predict the bioavailability of these compounds in plant roots.

Machine learning (ML) refers to the process by which computers learn patterns from data without the need for explicit programming [47]. Due to the precision and convergence advantages of supervised learning, ML has been extensively utilized in environmental science studies [48,49], including predicting the RCF of organic compounds. ML models are developed to account for various uptake processes [50,51], such as examining the RCF of a wide range of per- and polyfluoroalkyl substance compounds and investigating the critical threshold range for PFAS uptake [52]. Additionally, various ML algorithms have been employed to predict RCF across different crops, such as wheat and cabbage, while analyzing the effects of multiple factors to identify molar refractivity and molecular volume as key attribute descriptors for accurate prediction [44]. However, current research on ML models related to the bioavailability of plant roots has not sufficiently explored emerging contaminants, particularly aryl OPEs and PBDEs [53,54].

Moreover, most studies use Abraham molecular descriptors to characterize the properties of pollutants. However, Abraham descriptors have limitations, as they require experimental measurements for certain physicochemical properties, which can be challenging and time-consuming [55]. Molecular descriptors derived from ChemDes Version 3.2, which integrates multiple software packages and tools for descriptor calculation, provide a more comprehensive description of chemical structure. These descriptors have been successfully applied to predict various chemical and biological properties, such as mercury ecotoxicity and target protein interactions [52,56,57]. For example, GATSe8 is a geometric descriptor that captures the spatial arrangement of atoms within a molecule, offering insights into how molecular structure can influence environmental behavior. chiChain.3, a topological descriptor, is related to molecular connectivity, helping assess the stability and reactivity of a compound’s structure. Meanwhile, xlogP reflects a compound’s lipophilicity, indicating its affinity for lipid environments and serving as a predictor of bioaccumulation potential. Together, these descriptors provide a comprehensive view of a chemical’s behavior in an environmental matrix [57]. Given the complex chemical properties of both traditional and emerging ACs, incorporating a broader range of molecular descriptors is essential for conducting a more thorough analysis of their impact on ML model performance.

In general, ACs, including emerging compounds such as aryl OPEs and PBDEs, exhibit complex chemical structures and soil behaviors, making it difficult to assess their bioavailability and long-term impacts. The ML model allows for rapid prediction of these compounds’ behavior in soil–plant systems, eliminating the need for lengthy and complex laboratory tests. Therefore, this study aims to develop an ML model to predict the RCF of ACs in soil for edible plants. The model incorporates the chemical properties of traditional and emerging ACs (PAHs, PCBs, organochlorine pesticide, PBDEs, aryl OPEs, PAEs), molecular descriptors (e.g., GATSe8, chiChain.3, xlogP), soil–plant properties (SOM content, plant species, exposure time, protein content, lipid content), and cultivation modes. The model can be used to obtain RCF values for ACs without prior knowledge of their soil–plant behavior. Furthermore, a feature importance analysis was conducted to identify the key factors influencing the accumulation of traditional and emerging ACs in plant roots, providing valuable insights into their potential risks to soil health and food safety. The developed ML model facilitates the rapid assessment of ACs’ environmental behavior, which is crucial for managing contamination in agricultural ecosystems.

## 2. Materials and Methods

### 2.1. Dataset Collection

The dataset used in this study was sourced from the Web of Science database covering the period from 1967 to 2024. The search query employed was “aromatic pollutants” AND “plants or crops” AND “bioavailability or accumulation or uptake or translation” AND “soil”. The literature data were summarized based on key parameters, including SOM content, plant species, exposure time, protein content, lipid content, and cultivation mode. Each record in the dataset is represented by either field cultivation mode (1) or potted cultivation mode (0). In cases where the literature only provided soil organic carbon content without SOM content, the conversion factor of SOM content = soil organic carbon content * 1.724 was applied [58]. Studies lacking information on exposure time were excluded. Experimental data in tabular format, including bioconcentration factors (BCF) for plant roots, or RCF values were directly incorporated from the literature. For records where plant protein and lipid contents were not provided, values were supplemented using the US Department of Agriculture Food Ingredient Database (https://fdc.nal.usda.gov/index.html, accessed on 15 February 2024). In cases where specific plant exposure durations were missing, they were estimated based on the average growth period of the crops in the study region. The SMILE structural formula of organic compounds was obtained by querying the PubChem database (https://pubchem.ncbi.nlm.nih.gov/, accessed on 15 February 2024). The final dataset consisted of 878 data points, including 55 ACs (PAHs, PCBs, organochlorine pesticide, PBDEs, aryl OPEs, and PAEs) and 17 common edible plants (amaranth, cabbage, cape, carrot, Chinese cabbage, clove, leek, lemon, maize, onions, potato, pumpkin, radish, rice, ryegrass, turnips, and wheat). The numerical distribution of input and output features in the dataset is shown in Figure S1.

### 2.2. Selection of Molecular Descriptors

The studied ACs in the dataset were processed through the ChemDes platform, which generated 3679 molecular descriptors. Addressing multicollinearity among these descriptors is a standard practice in feature engineering. To prevent overfitting, Spearman correlation coefficients were used to assess the relationships between descriptors and logRCF. Data filtering was performed using the Pandas library, eliminating descriptors that were not significantly correlated with logRCF (*p* > 0.05) or had weak correlation (|R| ≤ 0.1). In cases of high correlation between descriptors (|R| > 0.8), those with poor correlation to logRCF were removed, retaining only the descriptors with strong correlation [52].

### 2.3. Machine Learning Models

The model utilizes a set of input variables, including selected molecular descriptors (Text S1), SOM content, plant culture mode, exposure time, protein content, and lipid content. The logRCF serves as the target predictive output (Table S1). Categorical features are converted using one-hot encoding. The data were normalized and randomly divided into training and test sets in an 8:2 ratio. To enhance model generalization, various models were constructed, including gradient boosting regression tree (GBRT), random forest (RF), support vector regression (SVR), and a regularized linear model (LR). The hyperparameters of GBRT, RF, and SVR were optimized using Bayesian optimization and five-fold cross-validation [59]. Boosting and bagging are two classic algorithms in ensemble learning [60]. GBRT is a boosting-based algorithm that iteratively reduces residuals from previous models to optimize in the direction of decreasing model residuals [61]. RF, a classic application of the bagging algorithm, combines multiple weak classifiers to improve accuracy and generalization [62]. SVM is a supervised learning model that can map low-dimensional input space data into high-dimensional data for linear separability, with SVR being a specific application of SVM used for fitting and predicting data. During SVR model processing, input features were standardized to maintain consistency in dimensions and units [63].

These methods were selected for their complementary strengths. GBRT was chosen for its ability to iteratively reduce residual errors and handle non-linear relationships in the data. RF offers robust performance in high-dimensional datasets by combining multiple decision trees, thereby improving accuracy through the bagging process. SVR excels at mapping data into higher-dimensional spaces to better capture non-linear relationships. Lasso regression applies L1 regularization to reduce overfitting, particularly in models with numerous variables, by shrinking less important coefficients to zero, which also aids in feature selection. Traditional multiple linear regression models are prone to overfitting when many variables are involved; however, Lasso regression with L1 regularization helps mitigate this risk [64]. The hyperparameters of GBRT, RF, and SVR were optimized using Bayesian optimization and five-fold cross-validation, which efficiently explores the hyperparameter space and ensures that the models generalize well to unseen data. The results of the model include the optimized hyperparameters (Table S2), predicted logRCF values (Table S3), and key performance indicators for model evaluation, as described in Section 2.4. The ML models were developed and validated using Jupyter Notebook (version 7.0.8), and the corresponding source code can be accessed in Text S2.

### 2.4. Model Validation

The performance of the logRCF prediction model is evaluated using three key parameters: coefficient of determination (R^2^), mean absolute error (MAE), and root mean square error (RMSE). R^2^ measures the level of agreement between the observed and predicted logRCF, with higher values closer to 1 indicating a better fit within the range of 0 to 1 [44]. MAE represents mean absolute error between the actual and predicted logRCF values [65]. RMSE quantifies the overall discrepancy between the observed and predicted values, calculated by taking the square root of the average of the squared differences [66].

$$R^{2}=1-\frac{\sum_{i}{\left(\hat{y_{i}}-y_{i}\right)}^{2}}{\sum_{i}{\left(\bar{y_{i}}-y_{i}\right)}^{2}}$$

$$MAE=\frac{1}{n}\sum_{i=1}^{n}\left|y_{i}-\hat{y_{i}}\right|$$

$$RMSE=\sqrt{\frac{1}{n}}\sum_{i=1}^{n}{\left(\hat{y_{i}}-y_{i}\right)}^{2}$$

where $\hat{y_{i}}$ represents the logRCF value of the i-th prediction, $y_{i}$ represents the logRCF value measured in the i-th experiment, and n represents the quantity.

### 2.5. Model Interpretability

**Figure 1:**
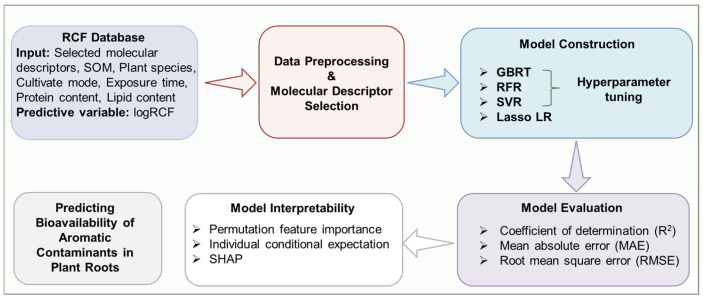
Workflow of model construction and evaluation.

While machine learning models such as GBRT and RF demonstrate strong performance, the lack of transparency in the process from input parameters to output results poses challenges for model interpretation [67]. This study explores several methods for interpreting these models. The primary approach used is perturbation analysis, which involves randomly replacing certain sample feature values with alternative values. By perturbing the input data and examining the variations in the model’s output before and after perturbation, the predictive behavior and underlying mechanisms of the complex model can be better elucidated. The analysis addresses both global and local perturbations [68]. In this study, model interpretability was enhanced through various methods to comprehensively analyze the impact of different variables. Global perturbations were analyzed using permutation feature importance (PFI), individual conditional expectation (ICE), and 3D interaction plots, while local perturbations were examined using the classic SHAP analysis. PFI, a model-independent approach, involves shuffling feature values to assess their impact on model performance, thereby identifying key features for prediction [69]. SHAP, based on the Shapley additive interpretation method, facilitates both global and local interpretations by calculating the marginal contribution of features to model output, providing valuable insights into model behavior [70]. The workflow of the machine learning model development and evaluation is shown in Figure 1.

$$Y_{j}=\sum_{i=1}^{n}\varphi_{j}(x_{i})$$

where $Y_{j}$ is the average SHAP value of the j-th feature, $\varphi_{j}$ is the SHAP value of the j-th feature of the i-th sample, and n is the number of samples.

## 3. Results and Discussion

### 3.1. t-Distributed Stochastic Neighbor Embedding (t-SNE) Plot of the RCF Dataset

**Figure 2:**
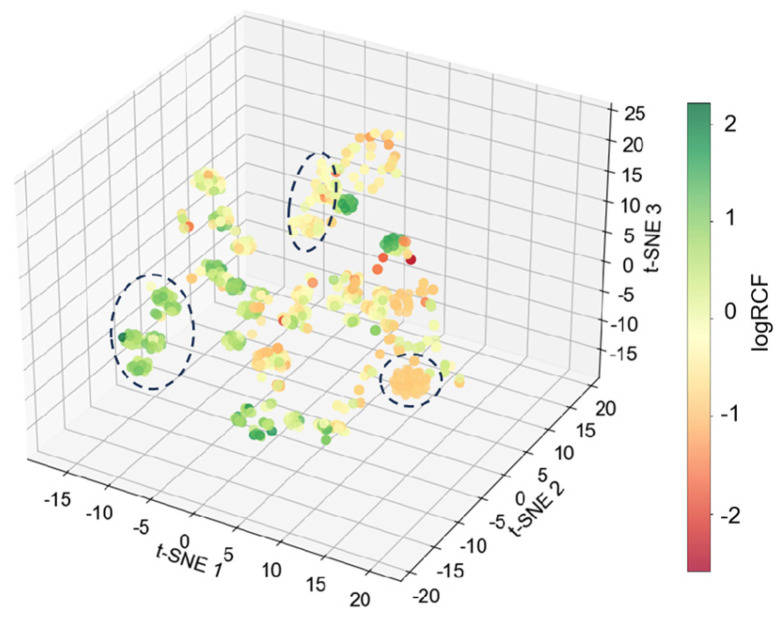
t-SNE visualization of RCF incorporating chemical, soil, and plant characteristics.

Unsupervised learning was employed to explore the dataset [71], with high-dimensional data visualized through dimensionality reduction to analyze the RCF dataset. t-SNE was used to create a feature space with reduced dimensions, where similar samples are represented by nearby points and dissimilar samples by more distant points [72]. As depicted in Figure 2, three distinct clusters were observed: PBDEs (orange part), PAHs (green part), and emerging ACs of aryl OPEs and PAEs (yellow part). These clusters effectively highlight the similarities among data points related to soil, plants, and chemicals, suggesting that t-SNE dimensionality reduction effectively captures key information about ACs entering the roots of crops and plants. Furthermore, the random distribution of clusters indicates that ACs with diverse physical and chemical properties are uniformly distributed within the t-SNE space.

### 3.2. logRCF Prediction with Machine Learning Models

**Figure 3:**
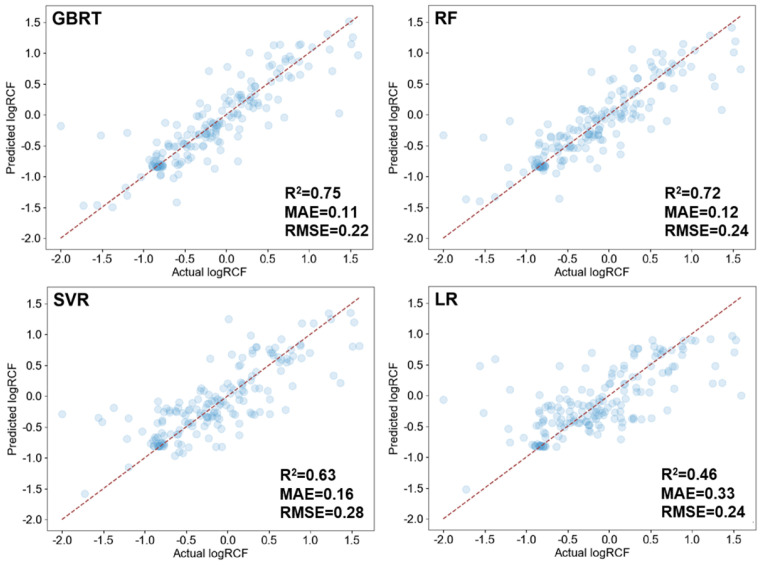
Performance of GBRT, RF, SVR, and LR models for predicting RCFs of traditional and emerging ACs.

Compared to linear models based on Lasso regression (R^2^ = 0.46, MAE = 0.33, RMSE = 0.24), ML models such as GBRT, RF, and SVR demonstrated superior performance (Figure 3). Among these, the GBRT model exhibited the highest performance (R^2^ = 0.75, MAE = 0.11, RMSE = 0.22), followed by the RF model (R^2^ = 0.72, MAE = 0.12, RMSE = 0.24), and SVR (R^2^ = 0.63, MAE = 0.16, and RMSE = 0.28). GBRT is widely used for predicting environmental pollutants, including emission degradation and accumulation in organisms. Its operation resembles that of RF, with both being tree-based decision models [73,74,75]. However, unlike RF, GBRT employs a gradient descent method to approximate residuals using the negative gradient of the loss function from the previous model [76], which explains its superior performance in this study. While SVR is effective with fewer features, it struggled with the complexities posed by numerous variables in this analysis [77]. Its performance is particularly sensitive to hyperparameter selection, which may have contributed to its less optimal results [78]. Based on these findings, the GBRT model was further employed to investigate the root accumulation of ACs. Table S2 presents the optimal hyperparameter values obtained from three different model grid searches.

### 3.3. Identification of Key Features and Their Influence on logRCF

**Figure 4:**
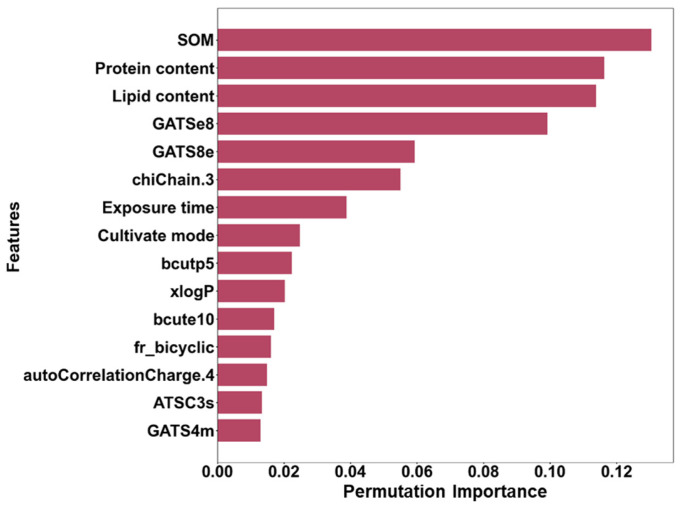
Permutation feature importance of the GBRT model for predicting RCFs of traditional and emerging ACs.

In the PFI analysis shown in Figure 4, the top 15 important features, including SOM, plant root protein content, root lipid content, exposure time, culture mode, and molecular descriptors, significantly influence the accumulation of ACs in plant roots. Notably, approximately 67% of these features are molecular descriptors, underscoring the pivotal role of molecular structure in plant root absorption. Among them, GATSe8 is a Geary autocorrelation descriptor that captures the electronegativity distribution pattern within a molecule at a specific lag (lag 8). ACs often exhibit delocalized electron systems, which strongly influence internal electronegativity. By weighting Sanderson atomic electronegativities, GATSe8 reveals how these patterns correlate over specific distances within the molecule, providing key insights into the unique interactions characteristic of aromatic structures [79]. The high importance of the fr_bicyclic descriptor indicates that the presence of a double-ring structure significantly influences the absorption of ACs by plant roots in soil. ChiChain 3 is a molecular descriptor that quantifies the degree of branching within a molecular chain. As a topological descriptor, it captures the complexity of molecular structures by emphasizing branching patterns [80].

SOM binds to ACs, thereby reducing their bioavailability and limiting their uptake by plant roots. However, under certain conditions, SOM can also enhance the absorption of specific ACs by altering their migration patterns. This dual functionality suggests that the role of SOM in pollutant accumulation is both complex and context-dependent. Protein content and lipid content also play significant roles. Proteins can selectively bind to particular ACs, thereby facilitating their accumulation in roots. In contrast, lipids exhibit strong adsorption capacity for hydrophobic ACs, such as PAHs, making it easier for these compounds to accumulate in lipid-rich root tissues. The importance of molecular descriptors underscores the critical influence of molecular structure on pollutant absorption. Descriptors such as GATSe8, chiChain.3, and fr_bicyclic reflect how the distribution of electronegativity and the complexity of molecular structures affect pollutants’ interactions with plant roots, ultimately influencing their uptake behavior.

**Figure 5:**
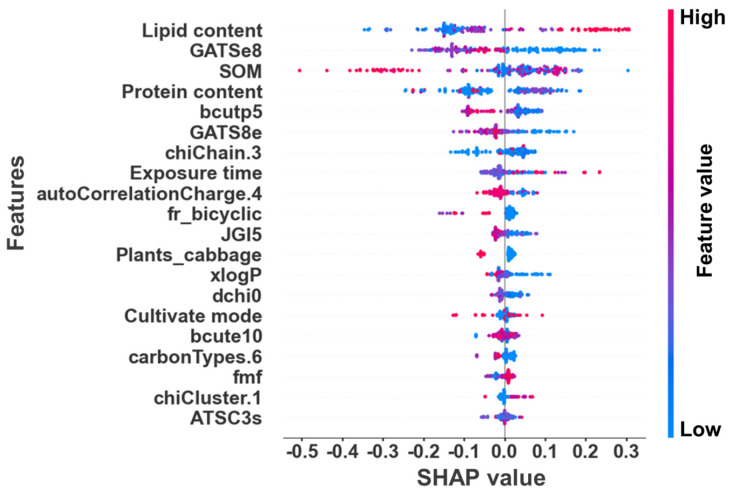
SHAP analysis of the GBRT model for predicting RCFs of traditional and emerging ACs.

Furthermore, the SHAP analysis provides additional insights into the specific contributions of these features to model predictions (Figure 5). The significant contributing features include lipid content, SOM, protein content, exposure time, cultivation mode, bcutp5, GATS8e, and xlogP. The high SHAP values associated with lipid content (represented by the red areas) suggest that roots with higher lipid concentrations are more likely to accumulate ACs, particularly lipophilic ones. SOM exhibits a combination of positive and negative SHAP values: at lower SOM levels, it promotes the migration and uptake of certain ACs, while at higher SOM concentrations, it restricts absorption by reducing ACs’ bioavailability. Similarly, SHAP values for protein content demonstrate both positive and negative effects, indicating that different ACs vary in their affinity for proteins. Some ACs enhance their absorption when bound to proteins, as transport mechanisms like H/phenanthrene co-transporters facilitate the uptake of low-molecular-weight PAHs into plant cells, thereby increasing their accumulation [81]. In contrast, the protein content in plant roots can inhibit certain compounds, such as aryl OPEs, from entering the plant’s vascular system by promoting their binding to the root cell wall, leading to reduced transport within the plant. Lastly, the SHAP values for exposure time indicate that longer exposure periods generally lead to higher accumulation, with mid-stage exposure showing particularly pronounced effects [82]. Each feature plays a multifaceted role in the model of pollutant accumulation in plant roots. These features not only directly impact pollutant absorption and transport but also indirectly regulate these processes by influencing plant and root growth, metabolic activity, and physicochemical environment.

Previous studies on RCF prediction have typically focused on traditional pollutants or specific plant species, which may limit the broad applicability of the model [35,83]. In this study, the developed ML model integrates both traditional and emerging ACs (e.g., aryl OPEs and PBDEs) across a wider range of edible plants, thereby expanding its applicability. Additionally, previous models, such as those used to predict the RCF of PFAS compounds or other persistent organic pollutants (e.g., PAHs and PCBs), have generally relied on a narrower set of molecular descriptors or specific environmental conditions, highlighting regular soil–plant system parameters such as SOM or root lipid content as key predictive features [44,84]. However, the ML model in this study incorporates a more diverse set of molecular descriptors (e.g., GATSe8, chiChain.3, fr_bicyclic, and xlogP), along with soil–plant properties (e.g., SOM content, plant species, and cultivation modes). As a result, more molecular features were identified as key influencing factors, allowing for a more comprehensive analysis of ACs’ behaviors in agricultural systems.

**Figure 6:**
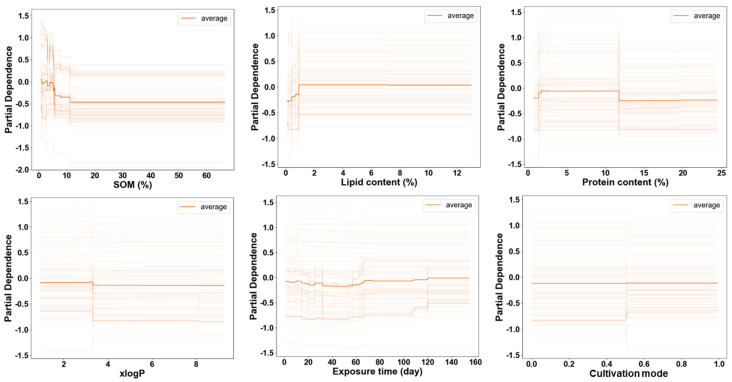
ICE analysis of key features of SOM, lipid content, protein content, xlogP, exposure time, and cultivation mode in the GBRT model for predicting RCFs of traditional and emerging ACs.

The ICE curve reveals the heterogeneous relationship between specific features and model predictions, illustrating how changes in these features affect the predictions for different individuals. Figure 6 highlights the average ICE plot for each feature, demonstrating varying degrees of dependency on logRCF values. The contribution and dependence of different variables on model predictions vary. In this model, certain variables, such as SOM and exposure time, exhibit strong dependencies within specific intervals, as evidenced by the more pronounced fluctuations in their curves in Figure 6. This indicates that these variables have a greater impact on the model’s prediction results, especially when SOM content is low and exposure time ranges from 40 to 80 days. SOM content emerges as a critical factor significantly influencing the absorption of ACs by plant roots. The figure indicates that RCF values decline notably as SOM content increases up to 10%, with a sharp drop observed between 4.9% and 5.7% SOM content, followed by stabilization. Some ACs have the ability to adsorb and bind to SOM, thereby affecting their uptake by plant roots [85,86,87]. Moreover, functional groups within SOM can interact with pollutants, influencing their mobility and transformation in soil, which indirectly impacts their uptake by plants [88].

Lipid content also plays an important role in the accumulation of ACs. An increase of up to 1% in plant root lipid content is positively correlated with logRCF values. Due to the presence of essential lipophilic components such as proteins and lipids within plant cells, some lipophilic ACs like PAHs can easily permeate plant root cells and accumulate within them [89]. Protein content in plants also significantly influences the absorption of ACs by the root system. A notable positive trend in logRCF values is observed as protein content increases within the range of 1.4% to 1.7%. However, this trend reverses around 11%. This shift may be due to varying affinities of different ACs for proteins; emerging ACs often exhibit high protein affinity, resulting in a positive relationship with protein content [90]. In contrast, traditional ACs such as PAHs and PCBs tend to bind more readily with lipids, potentially leading to a decrease in logRCF values as protein content increases [32,91].

The molecular descriptor xlogP is critical for evaluating the lipophilicity of pollutants [92]. As the xlogP value of pollutants increases, their absorption into plant roots generally decreases. This trend is due to higher xlogP values indicating greater solubility in lipid phases and lower solubility in aqueous phases, thereby reducing their bioavailability to plants.

Plant exposure time is another crucial factor, as illustrated in Figure 6, showing its dual effects. Initially, the absorption rate of ACs by plant roots tends to be higher during the early stages of exposure, driven by the large concentration gradient of pollutants. However, during long-term exposure, as plant roots grow and expand, a growth dilution effect may lead to decreased absorption rates. Studies have observed that the roots of maize (*Zea mays* L.) and peanut (*Arachis hypogaea* Linn.) rapidly absorb and accumulate PCBs and other ACs during the early vegetative growth stage. However, the concentrations of these compounds decline notably as plants progress to reproductive stages, likely due to reduced absorption capacity and growth-related dilution effects [39,93]. In contrast, the cultivation model of the plants does not exhibit a significant trend in ICE analysis. Plants cultivated under different conditions demonstrate varying physiological responses: potted plants typically exhibit more consistent physiological traits due to their stable environment [94], whereas outdoor plants must adapt to diverse environmental conditions, potentially affecting their ability to absorb and metabolize pollutants [95].

Among the methods used in this study, the machine learning model based on GBRT is identified as the best approach for accurately predicting logRCF values. The GBRT model excels in capturing complex, non-linear relationships between features and the target variable, offering high predictive performance. However, the t-SNE method plays a complementary role by providing valuable visual insights into the dataset, allowing us to explore clustering patterns and relationships that inform model development. Additionally, feature importance techniques like SHAP and PFI enhance the interpretability of the machine learning models, clarifying how individual features influence predictions.

## 4. Conclusions

ACs present in soil can be absorbed by plant roots, posing threats to ecological environments and human health. This study investigates root absorption by considering soil properties, plant characteristics, cultivation methods, and the molecular structures of both traditional and emerging ACs. A GBRT ML model was developed based on the dataset including 878 data points with 55 ACs to predict their RCFs, demonstrating strong predictive performance (R^2^ = 0.75, MAE = 0.11, RMSE = 0.22). SOM, protein content of plant root, and molecular descriptor xlogP were identified as crucial factors influencing RCF values. Notably, molecular descriptors related to the electronegativity distribution pattern (GATSe8) and double-ring structure (fr_bicyclic) of AC compounds were identified as key factors affecting RCFs for the first time. This study provides a comprehensive evaluation of AC root absorption in complex soil–plant systems, and offers valuable insights into the plant uptake and transport process of organic pollutants with aryl structures. Future work could enhance the performance of the prediction model by incorporating more advanced algorithms, such as deep learning, which may better capture complex environmental interactions. Additionally, expanding the dataset to include a wider range of molecular features and environmental variables could further improve the model’s predictive accuracy and generalizability.

## Data Availability

Data are contained within the article.

## Supplementary Materials

The following supporting information can be downloaded at https://www.mdpi.com/article/10.3390/toxics12100737/s1: Figure S1: Variable distribution of RCF dataset; Table S1: The dataset of absorption behaviors of aromatic contaminants in plant root [53,54,96,97,98,99,100,101,102,103,104,105,106,107,108,109,110,111]; Table S2: The optimal hyperparameter values for three different model grid searches; Table S3: Predicted logRCF values from different models; Text S1: Selected molecular descriptors; Text S2: The code of machine learning.
