# Supplementary material for: Machine Learning Models for Predicting Bioavailability of Traditional and Emerging Aromatic Contaminants in Plant Roots
Source: Toxics. 2024 Oct 12;12(10):737. doi: 10.3390/toxics12100737 (PMC11511036; doi:10.3390/toxics12100737)
Supplement: Supplementary file 1 [file toxics-12-00737-s001.zip › toxics-3227361-supplementary.pdf]

Supplementary Information

Machine Learning Models for Predicting Bioavailability of Traditional and Emerging Aromatic Contaminants in Plant Roots

Siyuan Li, Yuting Shen, Meng Gao, Huatai, Song, Zhanpeng Ge, Qiuyue Zhang, Jiaping Xu, Yu Wang*, Hongwen Sun*

MOE Key Laboratory of Pollution Processes and Environmental Criteria, College of Environmental Science and Engineering, Nankai University, Tianjin 300350, China

Corresponding Authors:

*Nankai University, 38 Tongyan Road, Jinnan District, Tianjin 300350, China. Tel.: +86 22 23509241.

E-mail: yu.wang@nankai.edu.cn (Y. Wang); sunhongwen@nankai.edu.cn (H. Sun)

Number of pages:52

Number of figure: 1

Number of tables: 3

Contents

Figure S1 Variable distribution of RCF dataset S3

Table S1 The dataset of absorption behaviors of aromatic contaminants in plant root S4

Table S2 The optimal hyperparameter values for three different model S40

Table S3 Predicted logRCF values from different models S41

Text S1 Selected molecular descriptors S46

Text S2 The code of machine learning S47

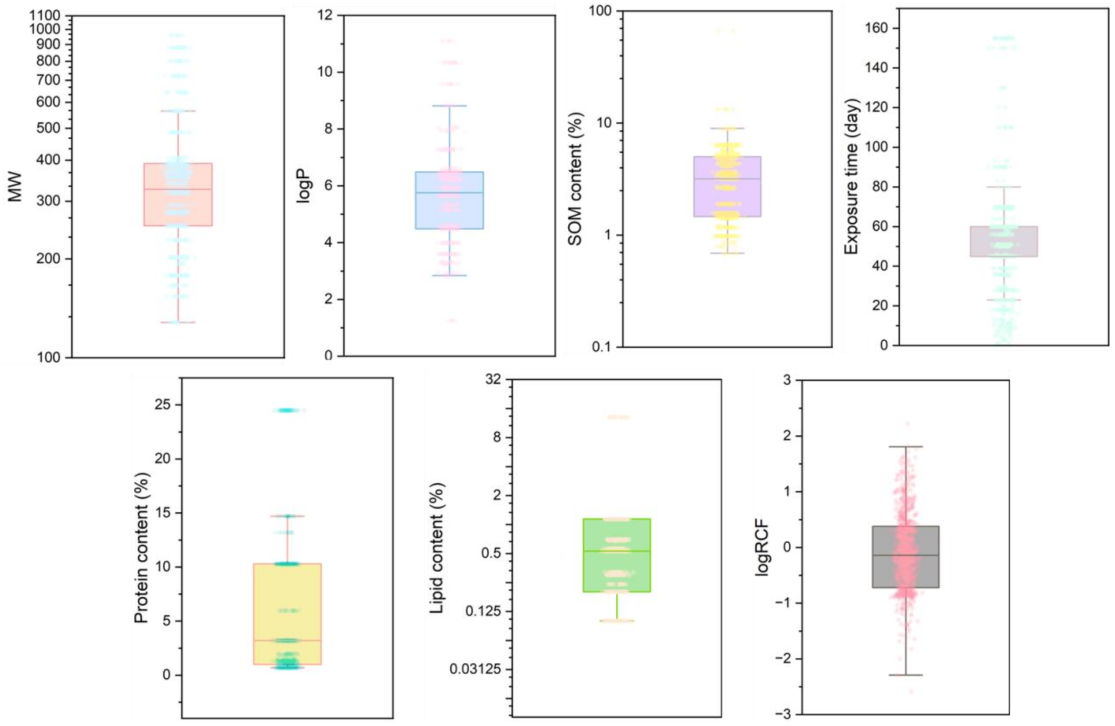

Figure S1 Variable distribution of RCF dataset

Table S1 The dataset of absorption behaviors of aromatic contaminants in plant root

**Compounds**	**Molecular Weight**	**SOM content/%**	**Plants**	**Cultivate mode**	**Exposure time (day)**	**Protein content (%)**	**Lipid content (%)**	**Log RCF**	**References**
Phenanthrene	178.23	0.79	Radish	0	64	0.7	0.1	-0.55	[96]
Fluorene	166.22	0.79	Radish	0	64	0.7	0.1	-0.10	[96]
Fluoranthene	202.25	0.79	Radish	0	64	0.7	0.1	0.58	[96]
Anthracene	178.23	0.79	Radish	0	64	0.7	0.1	-0.55	[96]
Dibutyl phthalate	390.6	0.79	Radish	0	64	0.7	0.1	-0.32	[96]
bis (2-ethylhexyl)phthalate	390.6	0.79	Radish	0	64	0.7	0.1	-1.52	[96]
Phenanthrene	178.23	1.45	Amaranth	0	45	13.2	0.32	-0.21	[97]
Phenanthrene	178.23	1.45	Amaranth	0	45	13.2	0.32	-0.57	[97]
Phenanthrene	178.23	1.45	Amaranth	0	45	13.2	0.32	-0.83	[97]
Phenanthrene	178.23	1.45	Amaranth	0	45	13.2	0.32	-0.86	[97]
Phenanthrene	178.23	1.45	Amaranth	0	45	13.2	0.32	-0.93	[97]
Phenanthrene	178.23	1.45	Amaranth	0	45	13.2	0.32	-0.87	[97]
Phenanthrene	178.23	1.45	Amaranth	0	45	13.2	0.32	-0.96	[97]
Pyrene	202.25	1.45	Amaranth	0	45	13.2	0.32	-0.83	[97]
Pyrene	202.25	1.45	Amaranth	0	45	13.2	0.32	-0.87	[97]
Pyrene	202.25	1.45	Amaranth	0	45	13.2	0.32	-0.99	[97]
Pyrene	202.25	1.45	Amaranth	0	45	13.2	0.32	-1.06	[97]
Pyrene	202.25	1.45	Amaranth	0	45	13.2	0.32	-1.09	[97]
Pyrene	202.25	1.45	Amaranth	0	45	13.2	0.32	-1.03	[97]
Pyrene	202.25	1.45	Amaranth	0	45	13.2	0.32	-1.08	[97]
Phenanthrene	178.23	1.45	Chinese cabbage	0	45	1.2	0.68	-0.57	[97]
Phenanthrene	178.23	1.45	Chinese cabbage	0	45	1.2	0.68	-0.54	[97]
Phenanthrene	178.23	1.45	Chinese	0	45	1.2	0.68	-0.55	[97]

Compounds	Molecular Weight	SOM content/%	Plants	Cultivate mode	Exposure time (day)	Protein content (%)	Lipid content (%)	Log RCF	References
			cabbage						
Phenanthrene	178.23	1.45	Chinese cabbage	0	45	1.2	0.68	-0.85	[97]
Phenanthrene	178.23	1.45	Chinese cabbage	0	45	1.2	0.68	-0.88	[97]
Phenanthrene	178.23	1.45	Chinese cabbage	0	45	1.2	0.68	-0.88	[97]
Phenanthrene	178.23	1.45	Chinese cabbage	0	45	1.2	0.68	-0.92	[97]
Phenanthrene	178.23	1.45	Chinese cabbage	0	45	1.2	0.68	-1.03	[97]
Pyrene	202.25	1.45	Chinese cabbage	0	45	1.2	0.68	-0.26	[97]
Pyrene	202.25	1.45	Chinese cabbage	0	45	1.2	0.68	-0.13	[97]
Pyrene	202.25	1.45	Chinese cabbage	0	45	1.2	0.68	-0.25	[97]
Pyrene	202.25	1.45	Chinese cabbage	0	45	1.2	0.68	-0.24	[97]
Pyrene	202.25	1.45	Chinese cabbage	0	45	1.2	0.68	-0.24	[97]
Pyrene	202.25	1.45	Chinese cabbage	0	45	1.2	0.68	-0.25	[97]
Pyrene	202.25	1.45	Chinese cabbage	0	45	1.2	0.68	-0.33	[97]
Pyrene	202.25	1.45	Chinese cabbage	0	45	1.2	0.68	-0.38	[97]

Compounds	Molecular Weight	SOM content/%	Plants	Cultivate mode	Exposure time (day)	Protein content (%)	Lipid content (%)	Log RCF	References
Phenanthrene	178.23	1.45	Ryegrass	0	45	24.5	0.32	-0.67	[97]
Phenanthrene	178.23	1.45	Ryegrass	0	45	24.5	0.32	-0.66	[97]
Phenanthrene	178.23	1.45	Ryegrass	0	45	24.5	0.32	-0.77	[97]
Phenanthrene	178.23	1.45	Ryegrass	0	45	24.5	0.32	-0.81	[97]
Phenanthrene	178.23	1.45	Ryegrass	0	45	24.5	0.32	-0.85	[97]
Phenanthrene	178.23	1.45	Ryegrass	0	45	24.5	0.32	-0.86	[97]
Phenanthrene	178.23	1.45	Ryegrass	0	45	24.5	0.32	-0.83	[97]
Phenanthrene	178.23	1.45	Ryegrass	0	45	24.5	0.32	-0.97	[97]
Pyrene	202.25	1.45	Ryegrass	0	45	24.5	0.32	-0.28	[97]
Pyrene	202.25	1.45	Ryegrass	0	45	24.5	0.32	-0.39	[97]
Pyrene	202.25	1.45	Ryegrass	0	45	24.5	0.32	-0.38	[97]
Pyrene	202.25	1.45	Ryegrass	0	45	24.5	0.32	-0.45	[97]
Pyrene	202.25	1.45	Ryegrass	0	45	24.5	0.32	-0.45	[97]
Pyrene	202.25	1.45	Ryegrass	0	45	24.5	0.32	-0.43	[97]
Pyrene	202.25	1.45	Ryegrass	0	45	24.5	0.32	-0.53	[97]
Pyrene	202.25	1.45	Ryegrass	0	45	24.5	0.32	-0.81	[97]
Acenaphthene	154.21	5.31	Wheat	0	45	10.3	1.14	0.56	[98]
Acenaphthene	154.21	1.41	Wheat	0	45	10.3	1.14	2.22	[98]
Acenaphthene	154.21	4.33	Wheat	0	45	10.3	1.14	0.89	[98]
Acenaphthene	154.21	2.70	Wheat	0	45	10.3	1.14	0.98	[98]
Acenaphthene	154.21	5.70	Wheat	0	45	10.3	1.14	0.32	[98]
Acenaphthene	154.21	2.60	Wheat	0	45	10.3	1.14	0.92	[98]
Acenaphthene	154.21	3.47	Wheat	0	45	10.3	1.14	0.48	[98]
Acenaphthene	154.21	5.30	Wheat	0	45	10.3	1.14	0.62	[98]
Acenaphthene	154.21	3.47	Wheat	0	45	10.3	1.14	0.54	[98]

Compounds	Molecular Weight	SOM content/%	Plants	Cultivate mode	Exposure time (day)	Protein content (%)	Lipid content (%)	Log RCF	References
Acenaphthene	154.21	6.22	Wheat	0	45	10.3	1.14	0.32	[98]
Acenaphthene	154.21	4.80	Wheat	0	45	10.3	1.14	0.69	[98]
Acenaphthene	154.21	3.47	Wheat	0	45	10.3	1.14	0.54	[98]
Acenaphthene	154.21	2.67	Wheat	0	45	10.3	1.14	0.83	[98]
Acenaphthene	154.21	4.74	Wheat	0	45	10.3	1.14	0.75	[98]
Acenaphthene	154.21	4.15	Wheat	0	45	10.3	1.14	0.61	[98]
Anthracene	178.23	5.31	Wheat	0	45	10.3	1.14	0.13	[98]
Anthracene	178.23	1.41	Wheat	0	45	10.3	1.14	0.78	[98]
Anthracene	178.23	4.33	Wheat	0	45	10.3	1.14	1.14	[98]
Anthracene	178.23	2.70	Wheat	0	45	10.3	1.14	0.76	[98]
Anthracene	178.23	5.70	Wheat	0	45	10.3	1.14	0.30	[98]
Anthracene	178.23	2.60	Wheat	0	45	10.3	1.14	0.50	[98]
Anthracene	178.23	3.47	Wheat	0	45	10.3	1.14	0.67	[98]
Anthracene	178.23	5.30	Wheat	0	45	10.3	1.14	0.61	[98]
Anthracene	178.23	3.47	Wheat	0	45	10.3	1.14	0.05	[98]
Anthracene	178.23	6.22	Wheat	0	45	10.3	1.14	0.34	[98]
Anthracene	178.23	4.80	Wheat	0	45	10.3	1.14	0.71	[98]
Anthracene	178.23	3.47	Wheat	0	45	10.3	1.14	0.05	[98]
Anthracene	178.23	2.67	Wheat	0	45	10.3	1.14	1.03	[98]
Anthracene	178.23	4.74	Wheat	0	45	10.3	1.14	0.65	[98]
Anthracene	178.23	4.15	Wheat	0	45	10.3	1.14	0.71	[98]
Fluoranthene	202.25	5.31	Wheat	0	45	10.3	1.14	0.30	[98]
Fluoranthene	202.25	1.41	Wheat	0	45	10.3	1.14	1.01	[98]
Fluoranthene	202.25	4.33	Wheat	0	45	10.3	1.14	0.57	[98]
Fluoranthene	202.25	2.70	Wheat	0	45	10.3	1.14	1.00	[98]

Compounds	Molecular Weight	SOM content/%	Plants	Cultivate mode	Exposure time (day)	Protein content (%)	Lipid content (%)	Log RCF	References
Fluoranthene	202.25	5.70	Wheat	0	45	10.3	1.14	0.55	[98]
Fluoranthene	202.25	2.60	Wheat	0	45	10.3	1.14	1.20	[98]
Fluoranthene	202.25	3.47	Wheat	0	45	10.3	1.14	0.74	[98]
Fluoranthene	202.25	5.30	Wheat	0	45	10.3	1.14	0.55	[98]
Fluoranthene	202.25	3.47	Wheat	0	45	10.3	1.14	0.50	[98]
Fluoranthene	202.25	6.22	Wheat	0	45	10.3	1.14	0.94	[98]
Fluoranthene	202.25	4.80	Wheat	0	45	10.3	1.14	1.30	[98]
Fluoranthene	202.25	3.47	Wheat	0	45	10.3	1.14	0.50	[98]
Fluoranthene	202.25	2.67	Wheat	0	45	10.3	1.14	1.04	[98]
Fluoranthene	202.25	4.74	Wheat	0	45	10.3	1.14	1.45	[98]
Fluoranthene	202.25	4.15	Wheat	0	45	10.3	1.14	1.51	[98]
Fluorene	166.22	5.31	Wheat	0	45	10.3	1.14	0.62	[98]
Fluorene	166.22	1.41	Wheat	0	45	10.3	1.14	0.96	[98]
Fluorene	166.22	4.33	Wheat	0	45	10.3	1.14	0.72	[98]
Fluorene	166.22	2.70	Wheat	0	45	10.3	1.14	1.14	[98]
Fluorene	166.22	5.70	Wheat	0	45	10.3	1.14	0.45	[98]
Fluorene	166.22	2.60	Wheat	0	45	10.3	1.14	1.11	[98]
Fluorene	166.22	3.47	Wheat	0	45	10.3	1.14	0.65	[98]
Fluorene	166.22	5.30	Wheat	0	45	10.3	1.14	0.60	[98]
Fluorene	166.22	3.47	Wheat	0	45	10.3	1.14	0.52	[98]
Fluorene	166.22	6.22	Wheat	0	45	10.3	1.14	0.34	[98]
Fluorene	166.22	4.80	Wheat	0	45	10.3	1.14	0.80	[98]
Fluorene	166.22	3.47	Wheat	0	45	10.3	1.14	0.52	[98]
Fluorene	166.22	2.67	Wheat	0	45	10.3	1.14	0.88	[98]
Fluorene	166.22	4.74	Wheat	0	45	10.3	1.14	-0.21	[98]

Compounds	Molecular Weight	SOM content/%	Plants	Cultivate mode	Exposure time (day)	Protein content (%)	Lipid content (%)	Log RCF	References
Fluorene	166.22	4.15	Wheat	0	45	10.3	1.14	0.70	[98]
Naphthalene	128.17	5.31	Wheat	0	45	10.3	1.14	0.58	[98]
Naphthalene	128.17	1.41	Wheat	0	45	10.3	1.14	0.99	[98]
Naphthalene	128.17	4.33	Wheat	0	45	10.3	1.14	0.84	[98]
Naphthalene	128.17	2.70	Wheat	0	45	10.3	1.14	1.17	[98]
Naphthalene	128.17	5.70	Wheat	0	45	10.3	1.14	0.39	[98]
Naphthalene	128.17	2.60	Wheat	0	45	10.3	1.14	0.98	[98]
Naphthalene	128.17	3.47	Wheat	0	45	10.3	1.14	0.34	[98]
Naphthalene	128.17	5.30	Wheat	0	45	10.3	1.14	0.63	[98]
Naphthalene	128.17	3.47	Wheat	0	45	10.3	1.14	0.35	[98]
Naphthalene	128.17	6.22	Wheat	0	45	10.3	1.14	0.18	[98]
Naphthalene	128.17	4.80	Wheat	0	45	10.3	1.14	0.56	[98]
Naphthalene	128.17	3.47	Wheat	0	45	10.3	1.14	0.35	[98]
Naphthalene	128.17	2.67	Wheat	0	45	10.3	1.14	0.83	[98]
Naphthalene	128.17	4.74	Wheat	0	45	10.3	1.14	0.83	[98]
Naphthalene	128.17	4.15	Wheat	0	45	10.3	1.14	0.90	[98]
Phenanthrene	178.23	5.31	Wheat	0	45	10.3	1.14	0.27	[98]
Phenanthrene	178.23	1.41	Wheat	0	45	10.3	1.14	0.84	[98]
Phenanthrene	178.23	4.33	Wheat	0	45	10.3	1.14	0.73	[98]
Phenanthrene	178.23	2.70	Wheat	0	45	10.3	1.14	0.86	[98]
Phenanthrene	178.23	5.70	Wheat	0	45	10.3	1.14	0.20	[98]
Phenanthrene	178.23	2.60	Wheat	0	45	10.3	1.14	0.86	[98]
Phenanthrene	178.23	3.47	Wheat	0	45	10.3	1.14	0.46	[98]
Phenanthrene	178.23	5.30	Wheat	0	45	10.3	1.14	0.44	[98]
Phenanthrene	178.23	3.47	Wheat	0	45	10.3	1.14	0.05	[98]

Compounds	Molecular Weight	SOM content/%	Plants	Cultivate mode	Exposure time (day)	Protein content (%)	Lipid content (%)	Log RCF	References
Phenanthrene	178.23	6.22	Wheat	0	45	10.3	1.14	0.28	[98]
Phenanthrene	178.23	4.80	Wheat	0	45	10.3	1.14	0.64	[98]
Phenanthrene	178.23	3.47	Wheat	0	45	10.3	1.14	0.05	[98]
Phenanthrene	178.23	2.67	Wheat	0	45	10.3	1.14	0.79	[98]
Phenanthrene	178.23	4.74	Wheat	0	45	10.3	1.14	0.53	[98]
Phenanthrene	178.23	4.15	Wheat	0	45	10.3	1.14	0.59	[98]
Pyrene	202.25	5.31	Wheat	0	45	10.3	1.14	-0.01	[98]
Pyrene	202.25	1.41	Wheat	0	45	10.3	1.14	0.87	[98]
Pyrene	202.25	4.33	Wheat	0	45	10.3	1.14	0.50	[98]
Pyrene	202.25	2.70	Wheat	0	45	10.3	1.14	1.06	[98]
Pyrene	202.25	5.70	Wheat	0	45	10.3	1.14	0.47	[98]
Pyrene	202.25	2.60	Wheat	0	45	10.3	1.14	1.14	[98]
Pyrene	202.25	3.47	Wheat	0	45	10.3	1.14	0.72	[98]
Pyrene	202.25	5.30	Wheat	0	45	10.3	1.14	0.40	[98]
Pyrene	202.25	3.47	Wheat	0	45	10.3	1.14	0.40	[98]
Pyrene	202.25	6.22	Wheat	0	45	10.3	1.14	0.47	[98]
Pyrene	202.25	4.80	Wheat	0	45	10.3	1.14	0.98	[98]
Pyrene	202.25	3.47	Wheat	0	45	10.3	1.14	0.40	[98]
Pyrene	202.25	2.67	Wheat	0	45	10.3	1.14	1.07	[98]
Pyrene	202.25	4.74	Wheat	0	45	10.3	1.14	0.70	[98]
Pyrene	202.25	4.15	Wheat	0	45	10.3	1.14	0.90	[98]
Benzo[a]anthracene	228.3	5.31	Wheat	0	45	10.3	1.14	-0.40	[98]
Benzo[a]anthracene	228.3	1.41	Wheat	0	45	10.3	1.14	1.11	[98]
Benzo[a]anthracene	228.3	4.33	Wheat	0	45	10.3	1.14	0.56	[98]
Benzo[a]anthracene	228.3	2.70	Wheat	0	45	10.3	1.14	1.03	[98]

Compounds	Molecular Weight	SOM content/%	Plants	Cultivate mode	Exposure time (day)	Protein content (%)	Lipid content (%)	Log RCF	References
Benzo[a]anthracene	228.3	5.70	Wheat	0	45	10.3	1.14	0.56	[98]
Benzo[a]anthracene	228.3	2.60	Wheat	0	45	10.3	1.14	0.96	[98]
Benzo[a]anthracene	228.3	3.47	Wheat	0	45	10.3	1.14	0.82	[98]
Benzo[a]anthracene	228.3	5.30	Wheat	0	45	10.3	1.14	0.28	[98]
Benzo[a]anthracene	228.3	3.47	Wheat	0	45	10.3	1.14	0.65	[98]
Benzo[a]anthracene	228.3	6.22	Wheat	0	45	10.3	1.14	0.76	[98]
Benzo[a]anthracene	228.3	4.80	Wheat	0	45	10.3	1.14	1.02	[98]
Benzo[a]anthracene	228.3	3.47	Wheat	0	45	10.3	1.14	0.65	[98]
Benzo[a]anthracene	228.3	2.67	Wheat	0	45	10.3	1.14	1.62	[98]
Benzo[a]anthracene	228.3	4.74	Wheat	0	45	10.3	1.14	0.99	[98]
Benzo[a]anthracene	228.3	4.15	Wheat	0	45	10.3	1.14	1.12	[98]
Benzo[a]pyrene	252.3	5.31	Wheat	0	45	10.3	1.14	-0.23	[98]
Benzo[a]pyrene	252.3	1.41	Wheat	0	45	10.3	1.14	1.08	[98]
Benzo[a]pyrene	252.3	4.33	Wheat	0	45	10.3	1.14	0.88	[98]
Benzo[a]pyrene	252.3	2.70	Wheat	0	45	10.3	1.14	1.05	[98]
Benzo[a]pyrene	252.3	5.70	Wheat	0	45	10.3	1.14	0.48	[98]
Benzo[a]pyrene	252.3	2.60	Wheat	0	45	10.3	1.14	1.34	[98]
Benzo[a]pyrene	252.3	3.47	Wheat	0	45	10.3	1.14	0.79	[98]
Benzo[a]pyrene	252.3	5.30	Wheat	0	45	10.3	1.14	0.52	[98]
Benzo[a]pyrene	252.3	3.47	Wheat	0	45	10.3	1.14	0.67	[98]
Benzo[a]pyrene	252.3	6.22	Wheat	0	45	10.3	1.14	0.80	[98]
Benzo[a]pyrene	252.3	4.80	Wheat	0	45	10.3	1.14	1.23	[98]
Benzo[a]pyrene	252.3	3.47	Wheat	0	45	10.3	1.14	0.67	[98]
Benzo[a]pyrene	252.3	2.67	Wheat	0	45	10.3	1.14	1.37	[98]
Benzo[a]pyrene	252.3	4.74	Wheat	0	45	10.3	1.14	0.82	[98]

Compounds	Molecular Weight	SOM content/%	Plants	Cultivate mode	Exposure time (day)	Protein content (%)	Lipid content (%)	Log RCF	References
Benzo[a]pyrene	252.3	4.15	Wheat	0	45	10.3	1.14	1.18	[98]
Benzo[b]fluoranthene	252.3	5.31	Wheat	0	45	10.3	1.14	-0.88	[98]
Benzo[b]fluoranthene	252.3	1.41	Wheat	0	45	10.3	1.14	0.46	[98]
Benzo[b]fluoranthene	252.3	4.33	Wheat	0	45	10.3	1.14	-0.16	[98]
Benzo[b]fluoranthene	252.3	2.70	Wheat	0	45	10.3	1.14	-0.10	[98]
Benzo[b]fluoranthene	252.3	5.70	Wheat	0	45	10.3	1.14	-0.50	[98]
Benzo[b]fluoranthene	252.3	2.60	Wheat	0	45	10.3	1.14	0.18	[98]
Benzo[b]fluoranthene	252.3	3.47	Wheat	0	45	10.3	1.14	0.19	[98]
Benzo[b]fluoranthene	252.3	5.30	Wheat	0	45	10.3	1.14	-0.25	[98]
Benzo[b]fluoranthene	252.3	3.47	Wheat	0	45	10.3	1.14	-0.29	[98]
Benzo[b]fluoranthene	252.3	6.22	Wheat	0	45	10.3	1.14	0.03	[98]
Benzo[b]fluoranthene	252.3	4.80	Wheat	0	45	10.3	1.14	0.24	[98]
Benzo[b]fluoranthene	252.3	3.47	Wheat	0	45	10.3	1.14	-0.29	[98]
Benzo[b]fluoranthene	252.3	2.67	Wheat	0	45	10.3	1.14	0.80	[98]
Benzo[b]fluoranthene	252.3	4.74	Wheat	0	45	10.3	1.14	-0.35	[98]
Benzo[b]fluoranthene	252.3	4.15	Wheat	0	45	10.3	1.14	0.34	[98]
Benzo[g,i,h]perylene	276.3	5.31	Wheat	0	45	10.3	1.14	-1.11	[98]
Benzo[g,i,h]perylene	276.3	1.41	Wheat	0	45	10.3	1.14	-0.31	[98]
Benzo[g,i,h]perylene	276.3	4.33	Wheat	0	45	10.3	1.14	-0.39	[98]
Benzo[g,i,h]perylene	276.3	2.70	Wheat	0	45	10.3	1.14	0.29	[98]
Benzo[g,i,h]perylene	276.3	5.70	Wheat	0	45	10.3	1.14	-0.04	[98]
Benzo[g,i,h]perylene	276.3	2.60	Wheat	0	45	10.3	1.14	0.70	[98]
Benzo[g,i,h]perylene	276.3	3.47	Wheat	0	45	10.3	1.14	0.00	[98]
Benzo[g,i,h]perylene	276.3	5.30	Wheat	0	45	10.3	1.14	-0.33	[98]
Benzo[g,i,h]perylene	276.3	3.47	Wheat	0	45	10.3	1.14	0.18	[98]

Compounds	Molecular Weight	SOM content/%	Plants	Cultivate mode	Exposure time (day)	Protein content (%)	Lipid content (%)	Log RCF	References
Benzo[g,i,h]perylene	276.3	6.22	Wheat	0	45	10.3	1.14	-0.23	[98]
Benzo[g,i,h]perylene	276.3	4.80	Wheat	0	45	10.3	1.14	0.47	[98]
Benzo[g,i,h]perylene	276.3	3.47	Wheat	0	45	10.3	1.14	0.18	[98]
Benzo[g,i,h]perylene	276.3	2.67	Wheat	0	45	10.3	1.14	0.58	[98]
Benzo[g,i,h]perylene	276.3	4.74	Wheat	0	45	10.3	1.14	-0.05	[98]
Benzo[g,i,h]perylene	276.3	4.15	Wheat	0	45	10.3	1.14	0.40	[98]
Benzo[k]fluoranthene	252.3	5.31	Wheat	0	45	10.3	1.14	-0.58	[98]
Benzo[k]fluoranthene	252.3	1.41	Wheat	0	45	10.3	1.14	-0.55	[98]
Benzo[k]fluoranthene	252.3	4.33	Wheat	0	45	10.3	1.14	-0.51	[98]
Benzo[k]fluoranthene	252.3	2.70	Wheat	0	45	10.3	1.14	0.85	[98]
Benzo[k]fluoranthene	252.3	5.70	Wheat	0	45	10.3	1.14	-0.25	[98]
Benzo[k]fluoranthene	252.3	2.60	Wheat	0	45	10.3	1.14	0.88	[98]
Benzo[k]fluoranthene	252.3	3.47	Wheat	0	45	10.3	1.14	0.03	[98]
Benzo[k]fluoranthene	252.3	5.30	Wheat	0	45	10.3	1.14	0.53	[98]
Benzo[k]fluoranthene	252.3	3.47	Wheat	0	45	10.3	1.14	-0.04	[98]
Benzo[k]fluoranthene	252.3	6.22	Wheat	0	45	10.3	1.14	-0.04	[98]
Benzo[k]fluoranthene	252.3	4.80	Wheat	0	45	10.3	1.14	0.79	[98]
Benzo[k]fluoranthene	252.3	3.47	Wheat	0	45	10.3	1.14	-0.04	[98]
Benzo[k]fluoranthene	252.3	2.67	Wheat	0	45	10.3	1.14	0.57	[98]
Benzo[k]fluoranthene	252.3	4.74	Wheat	0	45	10.3	1.14	1.05	[98]
Benzo[k]fluoranthene	252.3	4.15	Wheat	0	45	10.3	1.14	1.36	[98]
Chrysene	228.3	5.31	Wheat	0	45	10.3	1.14	-0.81	[98]
Chrysene	228.3	1.41	Wheat	0	45	10.3	1.14	-0.82	[98]
Chrysene	228.3	4.33	Wheat	0	45	10.3	1.14	-0.49	[98]
Chrysene	228.3	2.70	Wheat	0	45	10.3	1.14	-0.17	[98]

Compounds	Molecular Weight	SOM content/%	Plants	Cultivate mode	Exposure time (day)	Protein content (%)	Lipid content (%)	Log RCF	References
Chrysene	228.3	5.70	Wheat	0	45	10.3	1.14	-0.20	[98]
Chrysene	228.3	2.60	Wheat	0	45	10.3	1.14	-0.39	[98]
Chrysene	228.3	3.47	Wheat	0	45	10.3	1.14	-0.22	[98]
Chrysene	228.3	5.30	Wheat	0	45	10.3	1.14	-0.54	[98]
Chrysene	228.3	3.47	Wheat	0	45	10.3	1.14	-0.10	[98]
Chrysene	228.3	6.22	Wheat	0	45	10.3	1.14	-0.25	[98]
Chrysene	228.3	4.80	Wheat	0	45	10.3	1.14	0.09	[98]
Chrysene	228.3	3.47	Wheat	0	45	10.3	1.14	-0.10	[98]
Chrysene	228.3	2.67	Wheat	0	45	10.3	1.14	0.51	[98]
Chrysene	228.3	4.74	Wheat	0	45	10.3	1.14	0.24	[98]
Chrysene	228.3	4.15	Wheat	0	45	10.3	1.14	0.16	[98]
Dibenzo[a,h]anthracene	278.3	5.31	Wheat	0	45	10.3	1.14	-1.20	[98]
Dibenzo[a,h]anthracene	278.3	1.41	Wheat	0	45	10.3	1.14	-0.22	[98]
Dibenzo[a,h]anthracene	278.3	4.33	Wheat	0	45	10.3	1.14	0.88	[98]
Dibenzo[a,h]anthracene	278.3	2.70	Wheat	0	45	10.3	1.14	0.01	[98]
Dibenzo[a,h]anthracene	278.3	5.70	Wheat	0	45	10.3	1.14	-0.25	[98]
Dibenzo[a,h]anthracene	278.3	2.60	Wheat	0	45	10.3	1.14	-0.36	[98]
Dibenzo[a,h]anthracene	278.3	3.47	Wheat	0	45	10.3	1.14	0.10	[98]
Dibenzo[a,h]anthracene	278.3	5.30	Wheat	0	45	10.3	1.14	-0.65	[98]
Dibenzo[a,h]anthracene	278.3	3.47	Wheat	0	45	10.3	1.14	0.01	[98]
Dibenzo[a,h]anthracene	278.3	6.22	Wheat	0	45	10.3	1.14	-0.26	[98]
Dibenzo[a,h]anthracene	278.3	4.80	Wheat	0	45	10.3	1.14	0.40	[98]
Dibenzo[a,h]anthracene	278.3	3.47	Wheat	0	45	10.3	1.14	0.01	[98]
Dibenzo[a,h]anthracene	278.3	2.67	Wheat	0	45	10.3	1.14	0.64	[98]
Dibenzo[a,h]anthracene	278.3	4.74	Wheat	0	45	10.3	1.14	-0.14	[98]

Compounds	Molecular Weight	SOM content/%	Plants	Cultivate mode	Exposure time (day)	Protein content (%)	Lipid content (%)	Log RCF	References
Dibenzo[a,h]anthracene	278.3	4.15	Wheat	0	45	10.3	1.14	0.43	[98]
Phenanthrene	178.23	1.1-2.0	Carrot	1	45	0.9	0.24	0.50	[99]
Fluoranthene	202.25	1.1-2.0	Carrot	1	45	0.9	0.24	0.05	[99]
benzo[b]fluoranthene	252.3	1.1-2.0	Carrot	1	45	0.9	0.24	-0.92	[99]
benzo[k]fluoranthene	252.3	1.1-2.0	Carrot	1	45	0.9	0.24	-0.85	[99]
Anthracene	178.23	1.1-2.0	Carrot	1	45	0.9	0.24	0.15	[99]
Benzo[a]anthracene	228.3	1.1-2.0	Carrot	1	45	0.9	0.24	-0.77	[99]
Chrysene	228.3	1.1-2.0	Carrot	1	45	0.9	0.24	-1.64	[99]
Pyrene	202.25	1.1-2.0	Carrot	1	45	0.9	0.24	0.15	[99]
Benzo[a]pyrene	252.3	1.1-2.0	Carrot	1	45	0.9	0.24	-0.72	[99]
Benzo[e]pyrene	252.3	1.1-2.0	Carrot	1	45	0.9	0.24	-0.74	[99]
Indeno[1,2,3-cd]pyrene	276.3	1.1-2.0	Carrot	1	45	0.9	0.24	-0.80	[99]
Benz[phi]perylene	276.3	1.1-2.0	Carrot	1	45	0.9	0.24	-0.89	[99]
2,2',4,4',6-pentabromodiphenyl ether	564.7	3.19	Maize	0	60	3.2	0.53	-0.73	[100]
2,2',4,4',6-pentabromodiphenyl ether	564.7	1.90	Maize	0	60	3.2	0.53	-0.28	[100]
2,2',4,4',6-pentabromodiphenyl ether	564.7	0.98	Maize	0	60	3.2	0.53	-0.13	[100]
2,2',4,4',5,5'-Hexabromodiphenyl ether	643.6	1.90	Maize	0	60	3.2	0.53	-0.32	[100]
2,2',4,4',5,5'-Hexabromodiphenyl ether	643.6	0.98	Maize	0	60	3.2	0.53	-0.08	[100]
2,2',4,4',5,6'-Hexabromodiphenyl ether	643.6	3.19	Maize	0	60	3.2	0.53	-0.28	[100]
2,2',4,4',5,6'-Hexabromodiphenyl ether	643.6	1.90	Maize	0	60	3.2	0.53	-0.41	[100]
2,2',4,4',5,6'-Hexabromodiphenyl ether	643.6	0.98	Maize	0	60	3.2	0.53	-0.13	[100]
2,2',4-tribromodiphenyl ether	406.89	3.19	Maize	0	60	3.2	0.53	-0.15	[100]
2,2',4-tribromodiphenyl ether	406.89	1.90	Maize	0	60	3.2	0.53	-0.10	[100]
2,2',3,4,4',5',6-heptabromodiphenyl ether	722.5	3.19	Maize	0	60	3.2	0.53	-0.66	[100]
2,2',3,4,4',5',6-heptabromodiphenyl ether	722.5	1.90	Maize	0	60	3.2	0.53	-0.03	[100]

Compounds	Molecular Weight	SOM content/%	Plants	Cultivate mode	Exposure time (day)	Protein content (%)	Lipid content (%)	Log RCF	References
2,2',3,4,4',5',6-heptabromodiphenyl ether	722.5	0.98	Maize	0	60	3.2	0.53	-0.59	[100]
2,3,3',4,4',5',6-Heptabromodiphenyl ether	722.5	3.19	Maize	0	60	3.2	0.53	0.11	[100]
2,3,3',4,4',5',6-Heptabromodiphenyl ether	722.5	1.90	Maize	0	60	3.2	0.53	0.08	[100]
2,3,3',4,4',5',6-Heptabromodiphenyl ether	722.5	0.98	Maize	0	60	3.2	0.53	-0.11	[100]
2,2',3,3',4,4',5,6'-Octabromodiphenyl ether	801.4	3.19	Maize	0	60	3.2	0.53	-0.21	[100]
2,2',3,3',4,4',5,6'-Octabromodiphenyl ether	801.4	1.90	Maize	0	60	3.2	0.53	-0.43	[100]
2,2',3,3',4,4',5,6'-Octabromodiphenyl ether	801.4	0.98	Maize	0	60	3.2	0.53	-0.84	[100]
2,2',3,3',4,4',6,6'-Octabromodiphenyl ether	801.4	3.19	Maize	0	60	3.2	0.53	-0.45	[100]
2,2',3,3',4,4',6,6'-Octabromodiphenyl ether	801.4	1.90	Maize	0	60	3.2	0.53	-0.25	[100]
2,2',3,3',4,4',6,6'-Octabromodiphenyl ether	801.4	0.98	Maize	0	60	3.2	0.53	-0.49	[100]
Nonabromodiphenyl ether	880.3	3.19	Maize	0	60	3.2	0.53	-0.03	[100]
Nonabromodiphenyl ether	880.3	1.90	Maize	0	60	3.2	0.53	-1.15	[100]
Nonabromodiphenyl ether	880.3	0.98	Maize	0	60	3.2	0.53	-0.58	[100]
2,2',3,3',4,4',5,6,6'-nonabromodiphenyl ether	880.3	3.19	Maize	0	60	3.2	0.53	-0.56	[100]
2,2',3,3',4,4',5,6,6'-nonabromodiphenyl ether	880.3	1.90	Maize	0	60	3.2	0.53	-1.22	[100]
2,2',3,3',4,4',5,6,6'-nonabromodiphenyl ether	880.3	0.98	Maize	0	60	3.2	0.53	-0.81	[100]
1,2,3,4,5-Pentabromo-6-(2,3,5,6-tetrabromophenoxy)benzene	880.3	1.90	Maize	0	60	3.2	0.53	-1.20	[100]
1,2,3,4,5-Pentabromo-6-(2,3,5,6-tetrabromophenoxy)benzene	880.3	0.98	Maize	0	60	3.2	0.53	-1.37	[100]
Decabromodiphenyl ether	959.2	3.19	Maize	0	60	3.2	0.53	-0.55	[100]
Decabromodiphenyl ether	959.2	1.90	Maize	0	60	3.2	0.53	-0.61	[100]
Decabromodiphenyl ether	959.2	0.98	Maize	0	60	3.2	0.53	-0.77	[100]
2,4,4'-tribromodiphenyl ether	406.89	3.19	Maize	0	60	3.2	0.53	-0.20	[100]
2,4,4'-tribromodiphenyl ether	406.89	1.90	Maize	0	60	3.2	0.53	0.00	[100]
2,4,4'-tribromodiphenyl ether	406.89	0.98	Maize	0	60	3.2	0.53	-0.32	[100]
2,2',4,4'-Tetrabromodiphenyl ether	485.79	3.19	Maize	0	60	3.2	0.53	0.05	[100]

Compounds	Molecular Weight	SOM content/%	Plants	Cultivate mode	Exposure time (day)	Protein content (%)	Lipid content (%)	Log RCF	References
2,2',4,4'-Tetrabromodiphenyl ether	485.79	1.90	Maize	0	60	3.2	0.53	0.18	[100]
2,2',4,4'-Tetrabromodiphenyl ether	485.79	0.98	Maize	0	60	3.2	0.53	-0.12	[100]
2,2',4,5'-Tetrabromodiphenyl ether	485.79	3.19	Maize	0	60	3.2	0.53	-0.05	[100]
2,3',4,4'-tetrabromodiphenyl ether	485.79	3.19	Maize	0	60	3.2	0.53	0.09	[100]
2,3',4,4'-tetrabromodiphenyl ether	485.79	1.90	Maize	0	60	3.2	0.53	-0.35	[100]
2,3',4,4'-tetrabromodiphenyl ether	485.79	0.98	Maize	0	60	3.2	0.53	-0.84	[100]
2,2',3,4,4'-Pentabromodiphenyl ether	564.7	3.19	Maize	0	60	3.2	0.53	-0.08	[100]
2,2',3,4,4'-Pentabromodiphenyl ether	564.7	1.90	Maize	0	60	3.2	0.53	-0.47	[100]
2,2',3,4,4'-Pentabromodiphenyl ether	564.7	0.98	Maize	0	60	3.2	0.53	-0.17	[100]
2,2',4,4',5-Pentabromodiphenyl ether	564.7	3.19	Maize	0	60	3.2	0.53	-0.15	[100]
2,2',4,4',5-Pentabromodiphenyl ether	564.7	1.90	Maize	0	60	3.2	0.53	-0.13	[100]
2,2',4,4',5-Pentabromodiphenyl ether	564.7	0.98	Maize	0	60	3.2	0.53	-0.12	[100]
2,2',4,4',6-pentabromodiphenyl ether	564.7	3.19	Pumpkin	0	60	1	0.7	-0.47	[100]
2,2',4,4',6-pentabromodiphenyl ether	564.7	1.90	Pumpkin	0	60	1	0.7	-0.19	[100]
2,2',4,4',6-pentabromodiphenyl ether	564.7	0.98	Pumpkin	0	60	1	0.7	-0.09	[100]
2,2',4,4',5,5'-Hexabromodiphenyl ether	643.6	3.19	Pumpkin	0	60	1	0.7	-0.94	[100]
2,2',4,4',5,5'-Hexabromodiphenyl ether	643.6	1.90	Pumpkin	0	60	1	0.7	-0.47	[100]
2,2',4,4',5,5'-Hexabromodiphenyl ether	643.6	0.98	Pumpkin	0	60	1	0.7	-0.07	[100]
2,2',4,4',5,6'-Hexabromodiphenyl ether	643.6	3.19	Pumpkin	0	60	1	0.7	-0.30	[100]
2,2',4,4',5,6'-Hexabromodiphenyl ether	643.6	1.90	Pumpkin	0	60	1	0.7	-0.25	[100]
2,2',4,4',5,6'-Hexabromodiphenyl ether	643.6	0.98	Pumpkin	0	60	1	0.7	-0.17	[100]
2,2',4-tribromodiphenyl ether	406.89	3.19	Pumpkin	0	60	1	0.7	-0.11	[100]
2,2',4-tribromodiphenyl ether	406.89	1.90	Pumpkin	0	60	1	0.7	-0.12	[100]
2,2',4-tribromodiphenyl ether	406.89	0.98	Pumpkin	0	60	1	0.7	0.18	[100]
2,2',3,4,4',5',6-heptabromodiphenyl ether	722.5	3.19	Pumpkin	0	60	1	0.7	-0.57	[100]

Compounds	Molecular Weight	SOM content/%	Plants	Cultivate mode	Exposure time (day)	Protein content (%)	Lipid content (%)	Log RCF	References
2,2',3,4,4',5',6-heptabromodiphenyl ether	722.5	1.90	Pumpkin	0	60	1	0.7	-0.19	[100]
2,2',3,4,4',5',6-heptabromodiphenyl ether	722.5	0.98	Pumpkin	0	60	1	0.7	-0.72	[100]
2,3,3',4,4',5',6-Heptabromodiphenyl ether	722.5	3.19	Pumpkin	0	60	1	0.7	-0.07	[100]
2,3,3',4,4',5',6-Heptabromodiphenyl ether	722.5	1.90	Pumpkin	0	60	1	0.7	0.15	[100]
2,3,3',4,4',5',6-Heptabromodiphenyl ether	722.5	0.98	Pumpkin	0	60	1	0.7	-0.46	[100]
2,2',3,3',4,4',5,6'-Octabromodiphenyl ether	801.4	3.19	Pumpkin	0	60	1	0.7	0.21	[100]
2,2',3,3',4,4',5,6'-Octabromodiphenyl ether	801.4	1.90	Pumpkin	0	60	1	0.7	-0.09	[100]
2,2',3,3',4,4',6,6'-Octabromodiphenyl ether	801.4	3.19	Pumpkin	0	60	1	0.7	-0.45	[100]
2,2',3,3',4,4',6,6'-Octabromodiphenyl ether	801.4	1.90	Pumpkin	0	60	1	0.7	-0.10	[100]
2,2',3,3',4,4',6,6'-Octabromodiphenyl ether	801.4	0.98	Pumpkin	0	60	1	0.7	-0.34	[100]
Nonabromodiphenyl ether	880.3	3.19	Pumpkin	0	60	1	0.7	-0.14	[100]
Nonabromodiphenyl ether	880.3	1.90	Pumpkin	0	60	1	0.7	-1.39	[100]
Nonabromodiphenyl ether	880.3	0.98	Pumpkin	0	60	1	0.7	-0.76	[100]
2,2',3,3',4,4',5,6,6'-nonabromodiphenyl ether	880.3	3.19	Pumpkin	0	60	1	0.7	-0.28	[100]
2,2',3,3',4,4',5,6,6'-nonabromodiphenyl ether	880.3	1.90	Pumpkin	0	60	1	0.7	-1.62	[100]
2,2',3,3',4,4',5,6,6'-nonabromodiphenyl ether	880.3	0.98	Pumpkin	0	60	1	0.7	-1.03	[100]
1,2,3,4,5-Pentabromo-6-(2,3,5,6-tetrabromophenoxy)benzene	880.3	3.19	Pumpkin	0	60	1	0.7	-1.04	[100]
1,2,3,4,5-Pentabromo-6-(2,3,5,6-tetrabromophenoxy)benzene	880.3	1.90	Pumpkin	0	60	1	0.7	-1.18	[100]
1,2,3,4,5-Pentabromo-6-(2,3,5,6-tetrabromophenoxy)benzene	880.3	0.98	Pumpkin	0	60	1	0.7	-1.32	[100]
Decabromodiphenyl ether	959.2	3.19	Pumpkin	0	60	1	0.7	-0.30	[100]
Decabromodiphenyl ether	959.2	1.90	Pumpkin	0	60	1	0.7	-0.52	[100]
Decabromodiphenyl ether	959.2	0.98	Pumpkin	0	60	1	0.7	-0.68	[100]
2,4,4'-tribromodiphenyl ether	406.89	1.90	Pumpkin	0	60	1	0.7	-0.24	[100]
2,2',4,4'-Tetrabromodiphenyl ether	485.79	3.19	Pumpkin	0	60	1	0.7	0.18	[100]
2,2',4,4'-Tetrabromodiphenyl ether	485.79	1.90	Pumpkin	0	60	1	0.7	0.34	[100]

Compounds	Molecular Weight	SOM content/%	Plants	Cultivate mode	Exposure time (day)	Protein content (%)	Lipid content (%)	Log RCF	References
2,2',4,4'-Tetrabromodiphenyl ether	485.79	0.98	Pumpkin	0	60	1	0.7	0.06	[100]
2,2',4,5'-Tetrabromodiphenyl ether	485.79	3.19	Pumpkin	0	60	1	0.7	0.14	[100]
2,2',4,5'-Tetrabromodiphenyl ether	485.79	0.98	Pumpkin	0	60	1	0.7	-1.58	[100]
2,3',4,4'-tetrabromodiphenyl ether	485.79	3.19	Pumpkin	0	60	1	0.7	0.05	[100]
2,3',4,4'-tetrabromodiphenyl ether	485.79	1.90	Pumpkin	0	60	1	0.7	-0.21	[100]
2,3',4,4'-tetrabromodiphenyl ether	485.79	0.98	Pumpkin	0	60	1	0.7	-1.10	[100]
2,2',3,4,4'-Pentabromodiphenyl ether	564.7	3.19	Pumpkin	0	60	1	0.7	-0.19	[100]
2,2',3,4,4'-Pentabromodiphenyl ether	564.7	1.90	Pumpkin	0	60	1	0.7	-0.45	[100]
2,2',3,4,4'-Pentabromodiphenyl ether	564.7	0.98	Pumpkin	0	60	1	0.7	-0.16	[100]
2,2',4,4',5-Pentabromodiphenyl ether	564.7	3.19	Pumpkin	0	60	1	0.7	-0.10	[100]
2,2',4,4',5-Pentabromodiphenyl ether	564.7	1.90	Pumpkin	0	60	1	0.7	0.07	[100]
2,2',4,4',5-Pentabromodiphenyl ether	564.7	0.98	Pumpkin	0	60	1	0.7	0.00	[100]
2,2',4,4',6-pentabromodiphenyl ether	564.7	3.19	Ryegrass	0	60	24.5	0.56	-0.55	[100]
2,2',4,4',6-pentabromodiphenyl ether	564.7	1.90	Ryegrass	0	60	24.5	0.56	-0.42	[100]
2,2',4,4',6-pentabromodiphenyl ether	564.7	0.98	Ryegrass	0	60	24.5	0.56	0.03	[100]
2,2',4,4',5,5'-Hexabromodiphenyl ether	643.6	3.19	Ryegrass	0	60	24.5	0.56	-0.27	[100]
2,2',4,4',5,5'-Hexabromodiphenyl ether	643.6	1.90	Ryegrass	0	60	24.5	0.56	-0.32	[100]
2,2',4,4',5,5'-Hexabromodiphenyl ether	643.6	0.98	Ryegrass	0	60	24.5	0.56	-0.26	[100]
2,2',4,4',5,6'-Hexabromodiphenyl ether	643.6	1.90	Ryegrass	0	60	24.5	0.56	-0.45	[100]
2,2',4,4',5,6'-Hexabromodiphenyl ether	643.6	0.98	Ryegrass	0	60	24.5	0.56	-0.19	[100]
2,2',4-tribromodiphenyl ether	406.89	3.19	Ryegrass	0	60	24.5	0.56	-0.28	[100]
2,2',4-tribromodiphenyl ether	406.89	1.90	Ryegrass	0	60	24.5	0.56	-0.13	[100]
2,2',4-tribromodiphenyl ether	406.89	0.98	Ryegrass	0	60	24.5	0.56	-0.26	[100]
2,2',3,4,4',5',6-heptabromodiphenyl ether	722.5	3.19	Ryegrass	0	60	24.5	0.56	-0.41	[100]
2,2',3,4,4',5',6-heptabromodiphenyl ether	722.5	1.90	Ryegrass	0	60	24.5	0.56	-0.07	[100]

Compounds	Molecular Weight	SOM content/%	Plants	Cultivate mode	Exposure time (day)	Protein content (%)	Lipid content (%)	Log RCF	References
2,2',3,4,4',5',6-heptabromodiphenyl ether	722.5	0.98	Ryegrass	0	60	24.5	0.56	-0.54	[100]
2,3,3',4,4',5',6-Heptabromodiphenyl ether	722.5	3.19	Ryegrass	0	60	24.5	0.56	0.04	[100]
2,3,3',4,4',5',6-Heptabromodiphenyl ether	722.5	1.90	Ryegrass	0	60	24.5	0.56	-0.10	[100]
2,3,3',4,4',5',6-Heptabromodiphenyl ether	722.5	0.98	Ryegrass	0	60	24.5	0.56	-0.24	[100]
2,2',3,3',4,4',5,6'-Octabromodiphenyl ether	801.4	3.19	Ryegrass	0	60	24.5	0.56	0.14	[100]
2,2',3,3',4,4',5,6'-Octabromodiphenyl ether	801.4	1.90	Ryegrass	0	60	24.5	0.56	-0.66	[100]
2,2',3,3',4,4',5,6'-Octabromodiphenyl ether	801.4	0.98	Ryegrass	0	60	24.5	0.56	-0.87	[100]
2,2',3,3',4,4',6,6'-Octabromodiphenyl ether	801.4	3.19	Ryegrass	0	60	24.5	0.56	-0.24	[100]
2,2',3,3',4,4',6,6'-Octabromodiphenyl ether	801.4	1.90	Ryegrass	0	60	24.5	0.56	-0.54	[100]
2,2',3,3',4,4',6,6'-Octabromodiphenyl ether	801.4	0.98	Ryegrass	0	60	24.5	0.56	-0.72	[100]
Nonabromodiphenyl ether	880.3	3.19	Ryegrass	0	60	24.5	0.56	-0.64	[100]
Nonabromodiphenyl ether	880.3	1.90	Ryegrass	0	60	24.5	0.56	-1.82	[100]
Nonabromodiphenyl ether	880.3	0.98	Ryegrass	0	60	24.5	0.56	-0.74	[100]
2,2',3,3',4,4',5,6,6'-nonabromodiphenyl ether	880.3	3.19	Ryegrass	0	60	24.5	0.56	-0.64	[100]
2,2',3,3',4,4',5,6,6'-nonabromodiphenyl ether	880.3	1.90	Ryegrass	0	60	24.5	0.56	-1.49	[100]
2,2',3,3',4,4',5,6,6'-nonabromodiphenyl ether	880.3	0.98	Ryegrass	0	60	24.5	0.56	-0.97	[100]
1,2,3,4,5-Pentabromo-6-(2,3,5,6-tetrabromophenoxy)benzene	880.3	1.90	Ryegrass	0	60	24.5	0.56	-1.38	[100]
1,2,3,4,5-Pentabromo-6-(2,3,5,6-tetrabromophenoxy)benzene	880.3	0.98	Ryegrass	0	60	24.5	0.56	-1.45	[100]
Decabromodiphenyl ether	959.2	3.19	Ryegrass	0	60	24.5	0.56	-0.42	[100]
Decabromodiphenyl ether	959.2	1.90	Ryegrass	0	60	24.5	0.56	-0.69	[100]
Decabromodiphenyl ether	959.2	0.98	Ryegrass	0	60	24.5	0.56	-0.91	[100]
2,4,4'-tribromodiphenyl ether	406.89	1.90	Ryegrass	0	60	24.5	0.56	0.16	[100]
2,4,4'-tribromodiphenyl ether	406.89	0.98	Ryegrass	0	60	24.5	0.56	0.04	[100]
2,2',4,4'-Tetrabromodiphenyl ether	485.79	3.19	Ryegrass	0	60	24.5	0.56	0.07	[100]
2,2',4,4'-Tetrabromodiphenyl ether	485.79	1.90	Ryegrass	0	60	24.5	0.56	0.22	[100]

Compounds	Molecular Weight	SOM content/%	Plants	Cultivate mode	Exposure time (day)	Protein content (%)	Lipid content (%)	Log RCF	References
2,2',4,4'-Tetrabromodiphenyl ether	485.79	0.98	Ryegrass	0	60	24.5	0.56	-0.32	[100]
2,2',4,5'-Tetrabromodiphenyl ether	485.79	1.90	Ryegrass	0	60	24.5	0.56	-0.21	[100]
2,3',4,4'-tetrabromodiphenyl ether	485.79	3.19	Ryegrass	0	60	24.5	0.56	0.01	[100]
2,3',4,4'-tetrabromodiphenyl ether	485.79	1.90	Ryegrass	0	60	24.5	0.56	-0.28	[100]
2,3',4,4'-tetrabromodiphenyl ether	485.79	0.98	Ryegrass	0	60	24.5	0.56	-0.76	[100]
2,2',3,4,4'-Pentabromodiphenyl ether	564.7	3.19	Ryegrass	0	60	24.5	0.56	-0.29	[100]
2,2',3,4,4'-Pentabromodiphenyl ether	564.7	1.90	Ryegrass	0	60	24.5	0.56	-0.38	[100]
2,2',3,4,4'-Pentabromodiphenyl ether	564.7	0.98	Ryegrass	0	60	24.5	0.56	-0.10	[100]
2,2',4,4',5-Pentabromodiphenyl ether	564.7	3.19	Ryegrass	0	60	24.5	0.56	-0.16	[100]
2,2',4,4',5-Pentabromodiphenyl ether	564.7	1.90	Ryegrass	0	60	24.5	0.56	-0.31	[100]
2,2',4,4',5-Pentabromodiphenyl ether	564.7	0.98	Ryegrass	0	60	24.5	0.56	0.07	[100]
Dieldrin	380.9	1.40	Carrot	1	93	0.9	0.24	-0.60	[101]
Dieldrin	380.9	3.60	Carrot	1	93	0.9	0.24	-0.99	[101]
Dieldrin	380.9	66.50	Carrot	1	93	0.9	0.24	-2.29	[101]
Dieldrin	380.9	1.40	Onions	1	83	1.1	0.1	-1.68	[101]
Dieldrin	380.9	3.60	Onions	1	83	1.1	0.1	-1.73	[101]
Dieldrin	380.9	1.40	Radish	1	35	0.7	0.1	-1.38	[101]
Dieldrin	380.9	3.60	Radish	1	35	0.7	0.1	-1.33	[101]
Dieldrin	380.9	66.50	Radish	1	35	0.7	0.1	-2.59	[101]
Dieldrin	380.9	1.40	Turnips	1	120	0.9	0.1	-1.38	[101]
Dieldrin	380.9	3.60	Turnips	1	120	0.9	0.1	-1.56	[101]
Dieldrin	380.9	13.28	Maize	0	39	3.2	0.53	0.19	[102]
Dieldrin	380.9	0.86	Maize	0	39	3.2	0.53	1.47	[102]
Dieldrin	380.9	6.55	Maize	0	39	3.2	0.53	0.43	[102]
Dieldrin	380.9	1.20	Maize	0	39	3.2	0.53	1.29	[102]

Compounds	Molecular Weight	SOM content/%	Plants	Cultivate mode	Exposure time (day)	Protein content (%)	Lipid content (%)	Log RCF	References
Dieldrin	380.9	8.96	Maize	0	39	3.2	0.53	0.30	[102]
Dieldrin	380.9	0.69	Maize	0	39	3.2	0.53	1.52	[102]
Dieldrin	380.9	13.28	Maize	0	69	3.2	0.53	0.47	[102]
Dieldrin	380.9	0.86	Maize	0	69	3.2	0.53	1.38	[102]
Dieldrin	380.9	6.55	Maize	0	69	3.2	0.53	0.42	[102]
Dieldrin	380.9	1.20	Maize	0	69	3.2	0.53	1.30	[102]
Dieldrin	380.9	8.96	Maize	0	69	3.2	0.53	0.40	[102]
Dieldrin	380.9	0.69	Maize	0	69	3.2	0.53	1.55	[102]
Dieldrin	380.9	13.28	Maize	0	90	3.2	0.53	0.33	[102]
Dieldrin	380.9	0.86	Maize	0	90	3.2	0.53	1.35	[102]
Dieldrin	380.9	6.55	Maize	0	90	3.2	0.53	0.27	[102]
Dieldrin	380.9	1.20	Maize	0	90	3.2	0.53	1.40	[102]
Dieldrin	380.9	8.96	Maize	0	90	3.2	0.53	0.18	[102]
Dieldrin	380.9	0.69	Maize	0	90	3.2	0.53	1.33	[102]
Dieldrin	380.9	13.28	Maize	0	39	3.2	0.53	0.18	[102]
Dieldrin	380.9	0.86	Maize	0	39	3.2	0.53	1.63	[102]
Dieldrin	380.9	6.55	Maize	0	39	3.2	0.53	0.46	[102]
Dieldrin	380.9	1.20	Maize	0	39	3.2	0.53	1.22	[102]
Dieldrin	380.9	8.96	Maize	0	39	3.2	0.53	0.19	[102]
Dieldrin	380.9	0.69	Maize	0	39	3.2	0.53	1.62	[102]
Dieldrin	380.9	13.28	Maize	0	69	3.2	0.53	0.30	[102]
Dieldrin	380.9	0.86	Maize	0	69	3.2	0.53	1.46	[102]
Dieldrin	380.9	6.55	Maize	0	69	3.2	0.53	0.37	[102]
Dieldrin	380.9	1.20	Maize	0	69	3.2	0.53	1.24	[102]
Dieldrin	380.9	8.96	Maize	0	69	3.2	0.53	0.38	[102]

Compounds	Molecular Weight	SOM content/%	Plants	Cultivate mode	Exposure time (day)	Protein content (%)	Lipid content (%)	Log RCF	References
Dieldrin	380.9	0.69	Maize	0	69	3.2	0.53	1.28	[102]
Dieldrin	380.9	13.28	Maize	0	90	3.2	0.53	0.03	[102]
Dieldrin	380.9	0.86	Maize	0	90	3.2	0.53	1.59	[102]
Dieldrin	380.9	6.55	Maize	0	90	3.2	0.53	0.30	[102]
Dieldrin	380.9	1.20	Maize	0	90	3.2	0.53	1.22	[102]
Dieldrin	380.9	8.96	Maize	0	90	3.2	0.53	0.36	[102]
Dieldrin	380.9	0.69	Maize	0	90	3.2	0.53	1.48	[102]
Dieldrin	380.9	8.80	leek	0	39	1.5	0.1	-0.66	[103]
Dieldrin	380.9	8.80	leek	0	39	1.5	0.1	-0.89	[103]
Dieldrin	380.9	8.80	leek	0	39	1.5	0.1	-0.73	[103]
Mitotane	320	5.02	Radish	1	18	0.7	0.1	0.30	[53]
Mitotane	320	5.02	Radish	1	28	0.7	0.1	0.45	[53]
Mitotane	320	5.02	Radish	1	56	0.7	0.1	-0.08	[53]
Mitotane	320	5.02	Radish	1	23	0.7	0.1	0.02	[53]
Mitotane	320	5.02	Radish	1	36	0.7	0.1	-0.05	[53]
Mitotane	320	5.02	Radish	1	51	0.7	0.1	-0.14	[53]
Mitotane	320	5.02	Radish	1	70	0.7	0.1	0.45	[53]
Mitotane	320	6.36	Radish	1	18	0.7	0.1	-0.33	[53]
Mitotane	320	6.36	Radish	1	28	0.7	0.1	-0.26	[53]
Mitotane	320	6.36	Radish	1	56	0.7	0.1	-0.79	[53]
Mitotane	320	6.36	Radish	1	23	0.7	0.1	-0.70	[53]
Mitotane	320	6.36	Radish	1	36	0.7	0.1	-0.80	[53]
Mitotane	320	6.36	Radish	1	51	0.7	0.1	-0.46	[53]
Mitotane	320	6.36	Radish	1	70	0.7	0.1	-0.25	[53]
2,2-(2-Chlorophenyl-4'-chlorophenyl)-1,1-dichloroethene	318	5.02	Radish	1	18	0.7	0.1	0.02	[53]

Compounds	Molecular Weight	SOM content/%	Plants	Cultivate mode	Exposure time (day)	Protein content (%)	Lipid content (%)	Log RCF	References
2,2-(2-Chlorophenyl-4'-chlorophenyl)-1,1-dichloroethene	318	5.02	Radish	1	28	0.7	0.1	0.28	[53]
2,2-(2-Chlorophenyl-4'-chlorophenyl)-1,1-dichloroethene	318	5.02	Radish	1	56	0.7	0.1	-0.19	[53]
2,2-(2-Chlorophenyl-4'-chlorophenyl)-1,1-dichloroethene	318	5.02	Radish	1	23	0.7	0.1	-0.15	[53]
2,2-(2-Chlorophenyl-4'-chlorophenyl)-1,1-dichloroethene	318	5.02	Radish	1	36	0.7	0.1	-0.17	[53]
2,2-(2-Chlorophenyl-4'-chlorophenyl)-1,1-dichloroethene	318	5.02	Radish	1	51	0.7	0.1	-0.09	[53]
2,2-(2-Chlorophenyl-4'-chlorophenyl)-1,1-dichloroethene	318	5.02	Radish	1	70	0.7	0.1	0.42	[53]
2,2-(2-Chlorophenyl-4'-chlorophenyl)-1,1-dichloroethene	318	6.36	Radish	1	18	0.7	0.1	-0.37	[53]
2,2-(2-Chlorophenyl-4'-chlorophenyl)-1,1-dichloroethene	318	6.36	Radish	1	28	0.7	0.1	-0.31	[53]
2,2-(2-Chlorophenyl-4'-chlorophenyl)-1,1-dichloroethene	318	6.36	Radish	1	56	0.7	0.1	-0.77	[53]
2,2-(2-Chlorophenyl-4'-chlorophenyl)-1,1-dichloroethene	318	6.36	Radish	1	23	0.7	0.1	-0.53	[53]
2,2-(2-Chlorophenyl-4'-chlorophenyl)-1,1-dichloroethene	318	6.36	Radish	1	36	0.7	0.1	-1.00	[53]
2,2-(2-Chlorophenyl-4'-chlorophenyl)-1,1-dichloroethene	318	6.36	Radish	1	51	0.7	0.1	-0.62	[53]
2,2-(2-Chlorophenyl-4'-chlorophenyl)-1,1-dichloroethene	318	6.36	Radish	1	70	0.7	0.1	-0.49	[53]
1,1,1-trichloro-2-(2-chlorophenyl)-2-(4-chlorophenyl)ethane	354.5	5.02	Radish	1	18	0.7	0.1	-0.02	[53]
1,1,1-trichloro-2-(2-chlorophenyl)-2-(4-chlorophenyl)ethane	354.5	5.02	Radish	1	28	0.7	0.1	0.31	[53]
1,1,1-trichloro-2-(2-chlorophenyl)-2-(4-chlorophenyl)ethane	354.5	5.02	Radish	1	56	0.7	0.1	-0.25	[53]
1,1,1-trichloro-2-(2-chlorophenyl)-2-(4-chlorophenyl)ethane	354.5	5.02	Radish	1	23	0.7	0.1	-0.01	[53]
1,1,1-trichloro-2-(2-chlorophenyl)-2-(4-chlorophenyl)ethane	354.5	5.02	Radish	1	36	0.7	0.1	-0.21	[53]
1,1,1-trichloro-2-(2-chlorophenyl)-2-(4-chlorophenyl)ethane	354.5	5.02	Radish	1	51	0.7	0.1	-0.14	[53]
1,1,1-trichloro-2-(2-chlorophenyl)-2-(4-chlorophenyl)ethane	354.5	5.02	Radish	1	70	0.7	0.1	0.34	[53]
1,1,1-trichloro-2-(2-chlorophenyl)-2-(4-chlorophenyl)ethane	354.5	6.36	Radish	1	18	0.7	0.1	-0.22	[53]
1,1,1-trichloro-2-(2-chlorophenyl)-2-(4-chlorophenyl)ethane	354.5	6.36	Radish	1	28	0.7	0.1	-0.33	[53]
1,1,1-trichloro-2-(2-chlorophenyl)-2-(4-chlorophenyl)ethane	354.5	6.36	Radish	1	56	0.7	0.1	-0.79	[53]
1,1,1-trichloro-2-(2-chlorophenyl)-2-(4-chlorophenyl)ethane	354.5	6.36	Radish	1	23	0.7	0.1	-0.62	[53]
1,1,1-trichloro-2-(2-chlorophenyl)-2-(4-chlorophenyl)ethane	354.5	6.36	Radish	1	36	0.7	0.1	-0.84	[53]

Compounds	Molecular Weight	SOM content/%	Plants	Cultivate mode	Exposure time (day)	Protein content (%)	Lipid content (%)	Log RCF	References
1,1,1-trichloro-2-(2-chlorophenyl)-2-(4-chlorophenyl)ethane	354.5	6.36	Radish	1	51	0.7	0.1	-0.46	[53]
1,1,1-trichloro-2-(2-chlorophenyl)-2-(4-chlorophenyl)ethane	354.5	6.36	Radish	1	70	0.7	0.1	-0.30	[53]
Dichlorodiphenyldichloroethane	320	5.02	Radish	1	18	0.7	0.1	-0.23	[53]
Dichlorodiphenyldichloroethane	320	5.02	Radish	1	28	0.7	0.1	0.26	[53]
Dichlorodiphenyldichloroethane	320	5.02	Radish	1	56	0.7	0.1	0.00	[53]
Dichlorodiphenyldichloroethane	320	5.02	Radish	1	23	0.7	0.1	-0.09	[53]
Dichlorodiphenyldichloroethane	320	5.02	Radish	1	36	0.7	0.1	0.04	[53]
Dichlorodiphenyldichloroethane	320	5.02	Radish	1	51	0.7	0.1	0.14	[53]
Dichlorodiphenyldichloroethane	320	5.02	Radish	1	70	0.7	0.1	0.50	[53]
Dichlorodiphenyldichloroethane	320	6.36	Radish	1	18	0.7	0.1	-0.37	[53]
Dichlorodiphenyldichloroethane	320	6.36	Radish	1	28	0.7	0.1	-0.33	[53]
Dichlorodiphenyldichloroethane	320	6.36	Radish	1	56	0.7	0.1	-0.69	[53]
Dichlorodiphenyldichloroethane	320	6.36	Radish	1	23	0.7	0.1	-0.70	[53]
Dichlorodiphenyldichloroethane	320	6.36	Radish	1	36	0.7	0.1	-0.65	[53]
Dichlorodiphenyldichloroethane	320	6.36	Radish	1	51	0.7	0.1	-0.43	[53]
Dichlorodiphenyldichloroethane	320	6.36	Radish	1	70	0.7	0.1	-0.05	[53]
1,1-Dichloro-2,2-bis(4-chlorophenyl)ethene	318	5.02	Radish	1	18	0.7	0.1	-0.12	[53]
1,1-Dichloro-2,2-bis(4-chlorophenyl)ethene	318	5.02	Radish	1	28	0.7	0.1	0.25	[53]
1,1-Dichloro-2,2-bis(4-chlorophenyl)ethene	318	5.02	Radish	1	56	0.7	0.1	-0.16	[53]
1,1-Dichloro-2,2-bis(4-chlorophenyl)ethene	318	5.02	Radish	1	23	0.7	0.1	-0.09	[53]
1,1-Dichloro-2,2-bis(4-chlorophenyl)ethene	318	5.02	Radish	1	36	0.7	0.1	-0.21	[53]
1,1-Dichloro-2,2-bis(4-chlorophenyl)ethene	318	5.02	Radish	1	51	0.7	0.1	-0.05	[53]
1,1-Dichloro-2,2-bis(4-chlorophenyl)ethene	318	5.02	Radish	1	70	0.7	0.1	0.34	[53]
1,1-Dichloro-2,2-bis(4-chlorophenyl)ethene	318	6.36	Radish	1	18	0.7	0.1	-0.19	[53]
1,1-Dichloro-2,2-bis(4-chlorophenyl)ethene	318	6.36	Radish	1	28	0.7	0.1	-0.13	[53]

Compounds	Molecular Weight	SOM content/%	Plants	Cultivate mode	Exposure time (day)	Protein content (%)	Lipid content (%)	Log RCF	References
1,1-Dichloro-2,2-bis(4-chlorophenyl)ethene	318	6.36	Radish	1	56	0.7	0.1	-0.63	[53]
1,1-Dichloro-2,2-bis(4-chlorophenyl)ethene	318	6.36	Radish	1	23	0.7	0.1	-0.55	[53]
1,1-Dichloro-2,2-bis(4-chlorophenyl)ethene	318	6.36	Radish	1	36	0.7	0.1	-0.75	[53]
1,1-Dichloro-2,2-bis(4-chlorophenyl)ethene	318	6.36	Radish	1	51	0.7	0.1	-0.39	[53]
1,1-Dichloro-2,2-bis(4-chlorophenyl)ethene	318	6.36	Radish	1	70	0.7	0.1	-0.24	[53]
Chlorophenothane	354.5	5.02	Radish	1	18	0.7	0.1	-0.11	[53]
Chlorophenothane	354.5	5.02	Radish	1	28	0.7	0.1	0.16	[53]
Chlorophenothane	354.5	5.02	Radish	1	56	0.7	0.1	-0.38	[53]
Chlorophenothane	354.5	5.02	Radish	1	23	0.7	0.1	-0.08	[53]
Chlorophenothane	354.5	5.02	Radish	1	36	0.7	0.1	-0.15	[53]
Chlorophenothane	354.5	5.02	Radish	1	51	0.7	0.1	-0.09	[53]
Chlorophenothane	354.5	5.02	Radish	1	70	0.7	0.1	0.33	[53]
Chlorophenothane	354.5	6.36	Radish	1	18	0.7	0.1	-0.22	[53]
Chlorophenothane	354.5	6.36	Radish	1	28	0.7	0.1	-0.10	[53]
Chlorophenothane	354.5	6.36	Radish	1	56	0.7	0.1	-0.74	[53]
Chlorophenothane	354.5	6.36	Radish	1	23	0.7	0.1	-0.48	[53]
Chlorophenothane	354.5	6.36	Radish	1	36	0.7	0.1	-0.85	[53]
Chlorophenothane	354.5	6.36	Radish	1	51	0.7	0.1	-0.40	[53]
Chlorophenothane	354.5	6.36	Radish	1	70	0.7	0.1	-0.27	[53]
2,2′,4,5,5′-Pentachlorbiphenyl	326.4	5.02	Radish	1	18	0.7	0.1	0.81	[53]
2,4,5,2',5'-Pentachlorobiphenyl	326.4	5.02	Radish	1	28	0.7	0.1	0.49	[53]
2,2′,4,5,5′-Pentachlorbiphenyl	326.4	5.02	Radish	1	56	0.7	0.1	0.30	[53]
2,4,5,2',5'-Pentachlorobiphenyl	326.4	5.02	Radish	1	23	0.7	0.1	-0.08	[53]
2,2′,4,5,5′-Pentachlorbiphenyl	326.4	5.02	Radish	1	36	0.7	0.1	0.25	[53]
2,2′,4,5,5′-Pentachlorbiphenyl	326.4	5.02	Radish	1	51	0.7	0.1	-0.07	[53]

Compounds	Molecular Weight	SOM content/%	Plants	Cultivate mode	Exposure time (day)	Protein content (%)	Lipid content (%)	Log RCF	References
2,2′,4,5,5′-Pentachlorbiphenyl	326.4	5.02	Radish	1	70	0.7	0.1	0.79	[53]
2,4,5,2',5'-Pentachlorobiphenyl	326.4	6.36	Radish	1	18	0.7	0.1	-0.41	[53]
2,2′,4,5,5′-Pentachlorbiphenyl	326.4	6.36	Radish	1	28	0.7	0.1	-0.49	[53]
2,2′,4,5,5′-Pentachlorbiphenyl	326.4	6.36	Radish	1	56	0.7	0.1	-0.89	[53]
2,4,5,2',5'-Pentachlorobiphenyl	326.4	6.36	Radish	1	23	0.7	0.1	-0.73	[53]
2,2′,4,5,5′-Pentachlorbiphenyl	326.4	6.36	Radish	1	36	0.7	0.1	-1.13	[53]
2,2′,4,5,5′-Pentachlorbiphenyl	326.4	6.36	Radish	1	51	0.7	0.1	-0.88	[53]
2,2′,4,5,5′-Pentachlorbiphenyl	326.4	6.36	Radish	1	70	0.7	0.1	-0.72	[53]
2,2′,3,4,4′,5′-Hexachlorbiphenyl	360.9	5.02	Radish	1	18	0.7	0.1	0.39	[53]
2,2′,3,4,4′,5′-Hexachlorbiphenyl	360.9	5.02	Radish	1	28	0.7	0.1	0.27	[53]
2,2′,3,4,4′,5′-Hexachlorbiphenyl	360.9	5.02	Radish	1	56	0.7	0.1	-0.13	[53]
2,2′,3,4,4′,5′-Hexachlorbiphenyl	360.9	5.02	Radish	1	23	0.7	0.1	-0.16	[53]
2,2′,3,4,4′,5′-Hexachlorbiphenyl	360.9	5.02	Radish	1	36	0.7	0.1	0.13	[53]
2,2′,3,4,4′,5′-Hexachlorbiphenyl	360.9	5.02	Radish	1	51	0.7	0.1	-0.38	[53]
2,2′,3,4,4′,5′-Hexachlorbiphenyl	360.9	5.02	Radish	1	70	0.7	0.1	0.29	[53]
2,2′,3,4,4′,5′-Hexachlorbiphenyl	360.9	6.36	Radish	1	18	0.7	0.1	-0.47	[53]
2,2′,3,4,4′,5′-Hexachlorbiphenyl	360.9	6.36	Radish	1	28	0.7	0.1	-0.49	[53]
2,2′,3,4,4′,5′-Hexachlorbiphenyl	360.9	6.36	Radish	1	56	0.7	0.1	-1.09	[53]
2,2′,3,4,4′,5′-Hexachlorbiphenyl	360.9	6.36	Radish	1	23	0.7	0.1	-0.70	[53]
2,2′,3,4,4′,5′-Hexachlorbiphenyl	360.9	6.36	Radish	1	36	0.7	0.1	-1.01	[53]
2,2′,3,4,4′,5′-Hexachlorbiphenyl	360.9	6.36	Radish	1	51	0.7	0.1	-0.79	[53]
2,2′,3,4,4′,5′-Hexachlorbiphenyl	360.9	6.36	Radish	1	70	0.7	0.1	-0.43	[53]
2,2′,4,4′,5,5′-Hexachlorbiphenyl	360.9	5.02	Radish	1	18	0.7	0.1	0.55	[53]
2,2′,4,4′,5,5′-Hexachlorbiphenyl	360.9	5.02	Radish	1	28	0.7	0.1	0.25	[53]
2,2′,4,4′,5,5′-Hexachlorbiphenyl	360.9	5.02	Radish	1	56	0.7	0.1	0.06	[53]

Compounds	Molecular Weight	SOM content/%	Plants	Cultivate mode	Exposure time (day)	Protein content (%)	Lipid content (%)	Log RCF	References
2,2′,4,4′,5,5′-Hexachlorbiphenyl	360.9	5.02	Radish	1	23	0.7	0.1	-0.11	[53]
2,2′,4,4′,5,5′-Hexachlorbiphenyl	360.9	5.02	Radish	1	36	0.7	0.1	0.12	[53]
2,2′,4,4′,5,5′-Hexachlorbiphenyl	360.9	5.02	Radish	1	51	0.7	0.1	-0.33	[53]
2,2′,4,4′,5,5′-Hexachlorbiphenyl	360.9	5.02	Radish	1	70	0.7	0.1	0.51	[53]
2,2′,4,4′,5,5′-Hexachlorbiphenyl	360.9	6.36	Radish	1	18	0.7	0.1	-0.52	[53]
2,2′,4,4′,5,5′-Hexachlorbiphenyl	360.9	6.36	Radish	1	28	0.7	0.1	-0.57	[53]
2,2′,4,4′,5,5′-Hexachlorbiphenyl	360.9	6.36	Radish	1	56	0.7	0.1	-1.03	[53]
2,2′,4,4′,5,5′-Hexachlorbiphenyl	360.9	6.36	Radish	1	23	0.7	0.1	-0.77	[53]
2,2′,4,4′,5,5′-Hexachlorbiphenyl	360.9	6.36	Radish	1	36	0.7	0.1	-1.12	[53]
2,2′,4,4′,5,5′-Hexachlorbiphenyl	360.9	6.36	Radish	1	51	0.7	0.1	-0.80	[53]
2,2′,4,4′,5,5′-Hexachlorbiphenyl	360.9	6.36	Radish	1	70	0.7	0.1	-0.50	[53]
2,2′,3,4,4′,5,5′-Heptachlorbiphenyl	395.3	5.02	Radish	1	18	0.7	0.1	0.30	[53]
2,2′,3,4,4′,5,5′-Heptachlorbiphenyl	395.3	5.02	Radish	1	28	0.7	0.1	0.05	[53]
2,2′,3,4,4′,5,5′-Heptachlorbiphenyl	395.3	5.02	Radish	1	56	0.7	0.1	-0.12	[53]
2,2′,3,4,4′,5,5′-Heptachlorbiphenyl	395.3	5.02	Radish	1	23	0.7	0.1	-0.16	[53]
2,2′,3,4,4′,5,5′-Heptachlorbiphenyl	395.3	5.02	Radish	1	36	0.7	0.1	0.15	[53]
2,2′,3,4,4′,5,5′-Heptachlorbiphenyl	395.3	5.02	Radish	1	51	0.7	0.1	-0.36	[53]
2,2′,3,4,4′,5,5′-Heptachlorbiphenyl	395.3	5.02	Radish	1	70	0.7	0.1	0.23	[53]
2,2′,3,4,4′,5,5′-Heptachlorbiphenyl	395.3	6.36	Radish	1	18	0.7	0.1	-0.54	[53]
2,2′,3,4,4′,5,5′-Heptachlorbiphenyl	395.3	6.36	Radish	1	28	0.7	0.1	-0.60	[53]
2,2′,3,4,4′,5,5′-Heptachlorbiphenyl	395.3	6.36	Radish	1	56	0.7	0.1	-1.07	[53]
2,2′,3,4,4′,5,5′-Heptachlorbiphenyl	395.3	6.36	Radish	1	23	0.7	0.1	-0.74	[53]
2,2′,3,4,4′,5,5′-Heptachlorbiphenyl	395.3	6.36	Radish	1	36	0.7	0.1	-1.11	[53]
2,2′,3,4,4′,5,5′-Heptachlorbiphenyl	395.3	6.36	Radish	1	51	0.7	0.1	-0.80	[53]
2,2′,3,4,4′,5,5′-Heptachlorbiphenyl	395.3	6.36	Radish	1	70	0.7	0.1	-0.49	[53]

Compounds	Molecular Weight	SOM content/%	Plants	Cultivate mode	Exposure time (day)	Protein content (%)	Lipid content (%)	Log RCF	References
2,2′,5,5′-Tetrachlorbiphenyl	292	5.02	Radish	1	18	0.7	0.1	1.59	[53]
2,2′,5,5′-Tetrachlorbiphenyl	292	5.02	Radish	1	28	0.7	0.1	1.26	[53]
2,2′,5,5′-Tetrachlorbiphenyl	292	5.02	Radish	1	56	0.7	0.1	0.83	[53]
2,2′,5,5′-Tetrachlorbiphenyl	292	5.02	Radish	1	23	0.7	0.1	0.72	[53]
2,2′,5,5′-Tetrachlorbiphenyl	292	5.02	Radish	1	36	0.7	0.1	0.78	[53]
2,2′,5,5′-Tetrachlorbiphenyl	292	5.02	Radish	1	51	0.7	0.1	0.66	[53]
2,2′,5,5′-Tetrachlorbiphenyl	292	5.02	Radish	1	70	0.7	0.1	0.76	[53]
2,2′,5,5′-Tetrachlorbiphenyl	292	6.36	Radish	1	18	0.7	0.1	0.07	[53]
2,2′,5,5′-Tetrachlorbiphenyl	292	6.36	Radish	1	28	0.7	0.1	-0.19	[53]
2,2′,5,5′-Tetrachlorbiphenyl	292	6.36	Radish	1	56	0.7	0.1	0.04	[53]
2,2′,5,5′-Tetrachlorbiphenyl	292	6.36	Radish	1	23	0.7	0.1	-0.31	[53]
2,2′,5,5′-Tetrachlorbiphenyl	292	6.36	Radish	1	36	0.7	0.1	-0.14	[53]
2,2′,5,5′-Tetrachlorbiphenyl	292	6.36	Radish	1	51	0.7	0.1	-0.74	[53]
2,2′,5,5′-Tetrachlorbiphenyl	292	6.36	Radish	1	70	0.7	0.1	-0.22	[53]
Triphenylphosphate	326.3	4.33	Rice	1	10	14.7	0.3	-0.44	[104-108]
Triphenylphosphate	326.3	4.33	Rice	1	16	14.7	0.3	0.37	[104-108]
Triphenylphosphate	326.3	4.33	Rice	1	46	14.7	0.3	0.22	[104-108]
Triphenylphosphate	326.3	4.33	Rice	1	58	14.7	0.3	0.70	[104-108]
Triphenylphosphate	326.3	4.33	Rice	1	110	14.7	0.3	0.00	[104-108]
Triphenylphosphate	326.3	4.33	Rice	1	130	14.7	0.3	-1.00	[104-108]
Triphenylphosphate	326.3	4.33	Rice	1	150	14.7	0.3	-0.28	[104-108]
2-Ethylhexyl diphenyl phosphate	362.4	4.33	Rice	1	10	14.7	0.3	-0.02	[104-108]
2-Ethylhexyl diphenyl phosphate	362.4	4.33	Rice	1	16	14.7	0.3	-1.10	[104-108]
2-Ethylhexyl diphenyl phosphate	362.4	4.33	Rice	1	46	14.7	0.3	0.16	[104-108]
2-Ethylhexyl diphenyl phosphate	362.4	4.33	Rice	1	58	14.7	0.3	0.46	[104-108]

Compounds	Molecular Weight	SOM content/%	Plants	Cultivate mode	Exposure time (day)	Protein content (%)	Lipid content (%)	Log RCF	References
2-Ethylhexyl diphenyl phosphate	362.4	4.33	Rice	1	110	14.7	0.3	-0.38	[104-108]
2-Ethylhexyl diphenyl phosphate	362.4	4.33	Rice	1	130	14.7	0.3	0.42	[104-108]
2-Ethylhexyl diphenyl phosphate	362.4	4.33	Rice	1	150	14.7	0.3	0.29	[104-108]
Triphenyl phosphine oxide	278.3	4.33	Rice	1	10	14.7	0.3	-0.85	[104-108]
Triphenyl phosphine oxide	278.3	4.33	Rice	1	16	14.7	0.3	-1.22	[104-108]
Triphenyl phosphine oxide	278.3	4.33	Rice	1	46	14.7	0.3	-1.30	[104-108]
Triphenyl phosphine oxide	278.3	4.33	Rice	1	58	14.7	0.3	-0.18	[104-108]
Triphenyl phosphine oxide	278.3	4.33	Rice	1	110	14.7	0.3	-0.54	[104-108]
Triphenyl phosphine oxide	278.3	4.33	Rice	1	130	14.7	0.3	-0.59	[104-108]
Triphenyl phosphine oxide	278.3	4.33	Rice	1	150	14.7	0.3	-1.40	[104-108]
Tricresyl phosphate	368.4	4.33	Rice	1	10	14.7	0.3	-0.16	[104-108]
Tricresyl phosphate	368.4	4.33	Rice	1	16	14.7	0.3	-0.03	[104-108]
Tricresyl phosphate	368.4	4.33	Rice	1	46	14.7	0.3	-0.06	[104-108]
Tricresyl phosphate	368.4	4.33	Rice	1	58	14.7	0.3	-0.08	[104-108]
Tricresyl phosphate	368.4	4.33	Rice	1	110	14.7	0.3	0.13	[104-108]
Tricresyl phosphate	368.4	4.33	Rice	1	130	14.7	0.3	0.29	[104-108]
Tricresyl phosphate	368.4	4.33	Rice	1	150	14.7	0.3	-0.15	[104-108]
Triphenyl phosphate	326.3	3.69	Maize	1	20	3.2	0.53	0.01	[104-108]
Triphenyl phosphate	326.3	3.69	Maize	1	30	3.2	0.53	0.03	[104-108]
Triphenyl phosphate	326.3	3.69	Maize	1	50	3.2	0.53	-0.08	[104-108]
Triphenyl phosphate	326.3	3.69	Maize	1	80	3.2	0.53	-0.13	[104-108]
Triphenyl phosphate	326.3	3.69	Maize	1	110	3.2	0.53	1.36	[104-108]
Triphenyl phosphate	326.3	3.69	Maize	1	150	3.2	0.53	0.45	[104-108]
2-Ethylhexyl diphenyl phosphate	362.4	3.69	Maize	1	20	3.2	0.53	-0.54	[104-108]
2-Ethylhexyl diphenyl phosphate	362.4	3.69	Maize	1	30	3.2	0.53	-0.08	[104-108]

Compounds	Molecular Weight	SOM content/%	Plants	Cultivate mode	Exposure time (day)	Protein content (%)	Lipid content (%)	Log RCF	References
2-Ethylhexyl diphenyl phosphate	362.4	3.69	Maize	1	50	3.2	0.53	-0.55	[104-108]
2-Ethylhexyl diphenyl phosphate	362.4	3.69	Maize	1	80	3.2	0.53	-0.51	[104-108]
2-Ethylhexyl diphenyl phosphate	362.4	3.69	Maize	1	110	3.2	0.53	-0.77	[104-108]
2-Ethylhexyl diphenyl phosphate	362.4	3.69	Maize	1	150	3.2	0.53	0.15	[104-108]
Triphenyl-phosphine oxide	278.3	3.69	Maize	1	20	3.2	0.53	-0.51	[104-108]
Triphenyl-phosphine oxide	278.3	3.69	Maize	1	30	3.2	0.53	-0.55	[104-108]
Triphenyl-phosphine oxide	278.3	3.69	Maize	1	50	3.2	0.53	-0.48	[104-108]
Triphenyl-phosphine oxide	278.3	3.69	Maize	1	80	3.2	0.53	-2.00	[104-108]
Triphenyl-phosphine oxide	278.3	3.69	Maize	1	110	3.2	0.53	-0.60	[104-108]
Triphenyl-phosphine oxide	278.3	3.69	Maize	1	150	3.2	0.53	-0.02	[104-108]
Tricresyl phosphate	368.4	3.69	Maize	1	20	3.2	0.53	-0.72	[104-108]
Tricresyl phosphate	368.4	3.69	Maize	1	30	3.2	0.53	0.41	[104-108]
Tricresyl phosphate	368.4	3.69	Maize	1	50	3.2	0.53	-0.12	[104-108]
Tricresyl phosphate	368.4	3.69	Maize	1	80	3.2	0.53	-0.59	[104-108]
Tricresyl phosphate	368.4	3.69	Maize	1	110	3.2	0.53	-0.31	[104-108]
Tricresyl phosphate	368.4	3.69	Maize	1	150	3.2	0.53	-0.64	[104-108]
Di-n-butyl phthalate	278.3	1.57	cabbage	0	45	1.3	0.2	-0.80	[109]
Di-n-butyl phthalate	278.3	1.57	cabbage	0	45	1.3	0.2	-0.80	[109]
Di-n-butyl phthalate	278.3	1.57	cabbage	0	45	1.3	0.2	-0.80	[109]
Di-n-butyl phthalate	278.3	1.57	cabbage	0	45	1.3	0.2	-0.79	[109]
Di-n-butyl phthalate	278.3	1.57	cabbage	0	45	1.3	0.2	-0.83	[109]
Di-n-butyl phthalate	278.3	1.57	cabbage	0	45	1.3	0.2	-0.78	[109]
Di-n-butyl phthalate	278.3	1.57	cabbage	0	45	1.3	0.2	-0.87	[109]
Di-n-butyl phthalate	278.3	1.57	cabbage	0	45	1.3	0.2	-0.77	[109]
Di-n-butyl phthalate	278.3	1.57	cabbage	0	45	1.3	0.2	-0.85	[109]

Compounds	Molecular Weight	SOM content/%	Plants	Cultivate mode	Exposure time (day)	Protein content (%)	Lipid content (%)	Log RCF	References
Di-n-butyl phthalate	278.3	1.57	cabbage	0	45	1.3	0.2	-0.82	[109]
Di-n-butyl phthalate	278.3	1.57	cabbage	0	45	1.3	0.2	-0.82	[109]
Di-n-butyl phthalate	278.3	1.57	cabbage	0	45	1.3	0.2	-0.84	[109]
Di-n-butyl phthalate	278.3	1.57	cabbage	0	45	1.3	0.2	-0.84	[109]
Di-n-butyl phthalate	278.3	1.57	cabbage	0	45	1.3	0.2	-0.83	[109]
Di-n-butyl phthalate	278.3	1.57	cabbage	0	45	1.3	0.2	-0.83	[109]
Di-n-butyl phthalate	278.3	1.57	cabbage	0	45	1.3	0.2	-0.82	[109]
Di-n-butyl phthalate	278.3	1.57	cabbage	0	45	1.3	0.2	-0.85	[109]
Di-n-butyl phthalate	278.3	1.57	cabbage	0	45	1.3	0.2	-0.85	[109]
Di-n-butyl phthalate	278.3	1.57	cabbage	0	45	1.3	0.2	-0.84	[109]
Di-n-butyl phthalate	278.3	1.57	cabbage	0	45	1.3	0.2	-0.83	[109]
Di-n-butyl phthalate	278.3	1.57	cabbage	0	45	1.3	0.2	-0.88	[109]
Di-n-butyl phthalate	278.3	1.57	cabbage	0	45	1.3	0.2	-0.84	[109]
Di-n-butyl phthalate	278.3	1.57	cabbage	0	45	1.3	0.2	-0.86	[109]
Di-n-butyl phthalate	278.3	1.57	cabbage	0	45	1.3	0.2	-0.83	[109]
Di-n-butyl phthalate	278.3	1.57	cabbage	0	45	1.3	0.2	-0.87	[109]
Di-n-butyl phthalate	278.3	1.57	cabbage	0	45	1.3	0.2	-0.86	[109]
Di-n-butyl phthalate	278.3	1.57	cabbage	0	45	1.3	0.2	-0.85	[109]
Di-n-butyl phthalate	278.3	1.57	cabbage	0	45	1.3	0.2	-0.79	[109]
Di-n-butyl phthalate	278.3	1.57	cabbage	0	45	1.3	0.2	-0.85	[109]
Di-n-butyl phthalate	278.3	1.57	cabbage	0	45	1.3	0.2	-0.82	[109]
Di-n-butyl phthalate	278.3	1.57	cabbage	0	45	1.3	0.2	-0.88	[109]
Di-n-butyl phthalate	278.3	1.57	cabbage	0	45	1.3	0.2	-0.77	[109]
Di-n-butyl phthalate	278.3	1.57	cabbage	0	45	1.3	0.2	-0.84	[109]
Di-n-butyl phthalate	278.3	1.57	cabbage	0	45	1.3	0.2	-0.84	[109]

Compounds	Molecular Weight	SOM content/%	Plants	Cultivate mode	Exposure time (day)	Protein content (%)	Lipid content (%)	Log RCF	References
Di-n-butyl phthalate	278.3	1.57	cabbage	0	45	1.3	0.2	-0.84	[109]
Di-n-butyl phthalate	278.3	1.57	cabbage	0	45	1.3	0.2	-0.81	[109]
Di-n-butyl phthalate	278.3	1.57	cabbage	0	45	1.3	0.2	-0.89	[109]
Di-n-butyl phthalate	278.3	1.57	cabbage	0	45	1.3	0.2	-0.83	[109]
Di-n-butyl phthalate	278.3	1.57	cabbage	0	45	1.3	0.2	-0.88	[109]
Di-n-butyl phthalate	278.3	1.57	cabbage	0	45	1.3	0.2	-0.83	[109]
Di-n-butyl phthalate	278.3	1.57	cabbage	0	45	1.3	0.2	-0.90	[109]
Di-n-butyl phthalate	278.3	1.57	cabbage	0	45	1.3	0.2	-0.82	[109]
Di-n-butyl phthalate	278.3	1.57	cabbage	0	45	1.3	0.2	-0.88	[109]
Di-n-butyl phthalate	278.3	1.57	cabbage	0	45	1.3	0.2	-0.84	[109]
Di-n-butyl phthalate	278.3	1.57	cabbage	0	45	1.3	0.2	-0.87	[109]
Di-n-butyl phthalate	278.3	1.57	cabbage	0	45	1.3	0.2	-0.81	[109]
Di-n-butyl phthalate	278.3	1.57	cabbage	0	45	1.3	0.2	-0.83	[109]
Di-n-butyl phthalate	278.3	1.57	cabbage	0	45	1.3	0.2	-0.84	[109]
Di-n-butyl phthalate	278.3	1.57	cabbage	0	45	1.3	0.2	-0.87	[109]
Di-n-butyl phthalate	278.3	1.57	cabbage	0	45	1.3	0.2	-0.79	[109]
Di-n-butyl phthalate	278.3	1.57	cabbage	0	45	1.3	0.2	-0.91	[109]
Di-n-butyl phthalate	278.3	1.57	cabbage	0	45	1.3	0.2	-0.84	[109]
Di-(2-ethylhexyl) phthalate	390.6	1.57	cabbage	0	45	1.3	0.2	-0.85	[109]
Di-(2-ethylhexyl) phthalate	390.6	1.57	cabbage	0	45	1.3	0.2	-0.88	[109]
Di-(2-ethylhexyl) phthalate	390.6	1.57	cabbage	0	45	1.3	0.2	-0.83	[109]
Di-(2-ethylhexyl) phthalate	390.6	1.57	cabbage	0	45	1.3	0.2	-0.85	[109]
Di-(2-ethylhexyl) phthalate	390.6	1.57	cabbage	0	45	1.3	0.2	-0.84	[109]
Di-(2-ethylhexyl) phthalate	390.6	1.57	cabbage	0	45	1.3	0.2	-0.84	[109]
Di-(2-ethylhexyl) phthalate	390.6	1.57	cabbage	0	45	1.3	0.2	-0.86	[109]

Compounds	Molecular Weight	SOM content/%	Plants	Cultivate mode	Exposure time (day)	Protein content (%)	Lipid content (%)	Log RCF	References
Di-(2-ethylhexyl) phthalate	390.6	1.57	cabbage	0	45	1.3	0.2	-0.82	[109]
Di-(2-ethylhexyl) phthalate	390.6	1.57	cabbage	0	45	1.3	0.2	-0.84	[109]
Di-(2-ethylhexyl) phthalate	390.6	1.57	cabbage	0	45	1.3	0.2	-0.84	[109]
Di-(2-ethylhexyl) phthalate	390.6	1.57	cabbage	0	45	1.3	0.2	-0.83	[109]
Di-(2-ethylhexyl) phthalate	390.6	1.57	cabbage	0	45	1.3	0.2	-0.86	[109]
Di-(2-ethylhexyl) phthalate	390.6	1.57	cabbage	0	45	1.3	0.2	-0.84	[109]
Di-(2-ethylhexyl) phthalate	390.6	1.57	cabbage	0	45	1.3	0.2	-0.85	[109]
Di-(2-ethylhexyl) phthalate	390.6	1.57	cabbage	0	45	1.3	0.2	-0.84	[109]
Di-(2-ethylhexyl) phthalate	390.6	1.57	cabbage	0	45	1.3	0.2	-0.90	[109]
Di-(2-ethylhexyl) phthalate	390.6	1.57	cabbage	0	45	1.3	0.2	-0.83	[109]
Di-(2-ethylhexyl) phthalate	390.6	1.57	cabbage	0	45	1.3	0.2	-0.86	[109]
Di-(2-ethylhexyl) phthalate	390.6	1.57	cabbage	0	45	1.3	0.2	-0.82	[109]
Di-(2-ethylhexyl) phthalate	390.6	1.57	cabbage	0	45	1.3	0.2	-0.85	[109]
Di-(2-ethylhexyl) phthalate	390.6	1.57	cabbage	0	45	1.3	0.2	-0.80	[109]
Di-(2-ethylhexyl) phthalate	390.6	1.57	cabbage	0	45	1.3	0.2	-0.81	[109]
Di-(2-ethylhexyl) phthalate	390.6	1.57	cabbage	0	45	1.3	0.2	-0.78	[109]
Di-(2-ethylhexyl) phthalate	390.6	1.57	cabbage	0	45	1.3	0.2	-0.82	[109]
Di-(2-ethylhexyl) phthalate	390.6	1.57	cabbage	0	45	1.3	0.2	-0.77	[109]
Di-(2-ethylhexyl) phthalate	390.6	1.57	cabbage	0	45	1.3	0.2	-0.84	[109]
Di-(2-ethylhexyl) phthalate	390.6	1.57	cabbage	0	45	1.3	0.2	-0.83	[109]
Di-(2-ethylhexyl) phthalate	390.6	1.57	cabbage	0	45	1.3	0.2	-0.82	[109]
Di-(2-ethylhexyl) phthalate	390.6	1.57	cabbage	0	45	1.3	0.2	-0.79	[109]
Di-(2-ethylhexyl) phthalate	390.6	1.57	cabbage	0	45	1.3	0.2	-0.85	[109]
Di-(2-ethylhexyl) phthalate	390.6	1.57	cabbage	0	45	1.3	0.2	-0.77	[109]
Di-(2-ethylhexyl) phthalate	390.6	1.57	cabbage	0	45	1.3	0.2	-0.83	[109]

Compounds	Molecular Weight	SOM content/%	Plants	Cultivate mode	Exposure time (day)	Protein content (%)	Lipid content (%)	Log RCF	References
Di-(2-ethylhexyl) phthalate	390.6	1.57	cabbage	0	45	1.3	0.2	-0.77	[109]
Di-(2-ethylhexyl) phthalate	390.6	1.57	cabbage	0	45	1.3	0.2	-0.79	[109]
Di-(2-ethylhexyl) phthalate	390.6	1.57	cabbage	0	45	1.3	0.2	-0.77	[109]
Di-(2-ethylhexyl) phthalate	390.6	1.57	cabbage	0	45	1.3	0.2	-0.84	[109]
Di-(2-ethylhexyl) phthalate	390.6	1.57	cabbage	0	45	1.3	0.2	-0.79	[109]
Di-(2-ethylhexyl) phthalate	390.6	1.57	cabbage	0	45	1.3	0.2	-0.87	[109]
Di-(2-ethylhexyl) phthalate	390.6	1.57	cabbage	0	45	1.3	0.2	-0.80	[109]
Di-(2-ethylhexyl) phthalate	390.6	1.57	cabbage	0	45	1.3	0.2	-0.89	[109]
Di-(2-ethylhexyl) phthalate	390.6	1.57	cabbage	0	45	1.3	0.2	-0.78	[109]
Di-(2-ethylhexyl) phthalate	390.6	1.57	cabbage	0	45	1.3	0.2	-0.80	[109]
Di-(2-ethylhexyl) phthalate	390.6	1.57	cabbage	0	45	1.3	0.2	-0.82	[109]
Di-(2-ethylhexyl) phthalate	390.6	1.57	cabbage	0	45	1.3	0.2	-0.79	[109]
Di-(2-ethylhexyl) phthalate	390.6	1.57	cabbage	0	45	1.3	0.2	-0.83	[109]
Di-(2-ethylhexyl) phthalate	390.6	1.57	cabbage	0	45	1.3	0.2	-0.89	[109]
Di-(2-ethylhexyl) phthalate	390.6	1.57	cabbage	0	45	1.3	0.2	-0.79	[109]
Di-(2-ethylhexyl) phthalate	390.6	1.57	cabbage	0	45	1.3	0.2	-0.83	[109]
Di-(2-ethylhexyl) phthalate	390.6	1.57	cabbage	0	45	1.3	0.2	-0.80	[109]
Di-(2-ethylhexyl) phthalate	390.6	1.57	cabbage	0	45	1.3	0.2	-0.84	[109]
Di-(2-ethylhexyl) phthalate	390.6	1.57	cabbage	0	45	1.3	0.2	-0.83	[109]
Di-(2-ethylhexyl) phthalate	390.6	1.57	cabbage	0	45	1.3	0.2	-0.90	[109]
Di-n-butyl phthalate	278.3	1.17	Maize	1	155	3.2	0.53	0.18	[110]
Di-n-butyl phthalate	278.3	1.17	Maize	1	155	3.2	0.53	0.06	[110]
Di-n-butyl phthalate	278.3	1.17	Maize	1	155	3.2	0.53	0.31	[110]
Di-n-butyl phthalate	278.3	1.17	Maize	1	155	3.2	0.53	0.26	[110]
Di-(2-ethylhexyl) phthalate	390.6	1.17	Maize	1	155	3.2	0.53	0.19	[110]

Compounds	Molecular Weight	SOM content/%	Plants	Cultivate mode	Exposure time (day)	Protein content (%)	Lipid content (%)	Log RCF	References
Di-(2-ethylhexyl) phthalate	390.6	1.17	Maize	1	155	3.2	0.53	0.14	[110]
Di-(2-ethylhexyl) phthalate	390.6	1.17	Maize	1	155	3.2	0.53	0.30	[110]
Di-(2-ethylhexyl) phthalate	390.6	1.17	Maize	1	155	3.2	0.53	0.32	[110]
Diisobutyl phthalate	278.34	1.17	Maize	1	155	3.2	0.53	0.32	[110]
Diisobutyl phthalate	278.34	1.17	Maize	1	155	3.2	0.53	0.22	[110]
Diisobutyl phthalate	278.34	1.17	Maize	1	155	3.2	0.53	0.48	[110]
Diisobutyl phthalate	278.34	1.17	Maize	1	155	3.2	0.53	0.40	[110]
Dimethyl phthalate	194.18	1.17	Maize	1	155	3.2	0.53	0.18	[110]
Dimethyl phthalate	194.18	1.17	Maize	1	155	3.2	0.53	0.27	[110]
Dimethyl phthalate	194.18	1.17	Maize	1	155	3.2	0.53	0.29	[110]
Dimethyl phthalate	194.18	1.17	Maize	1	155	3.2	0.53	0.34	[110]
Di-n-butyl phthalate	278.3	1.17	Potato	1	155	2	0.1	-0.31	[110]
Di-n-butyl phthalate	278.3	1.17	Potato	1	155	2	0.1	-0.25	[110]
Di-n-butyl phthalate	278.3	1.17	Potato	1	155	2	0.1	-0.09	[110]
Di-n-butyl phthalate	278.3	1.17	Potato	1	155	2	0.1	-0.25	[110]
Di(2-Ethylhexyl) phthalate	390.6	1.17	Potato	1	155	2	0.1	0.08	[110]
Di(2-Ethylhexyl) phthalate	390.6	1.17	Potato	1	155	2	0.1	0.07	[110]
Di(2-Ethylhexyl) phthalate	390.6	1.17	Potato	1	155	2	0.1	-0.06	[110]
Di(2-Ethylhexyl) phthalate	390.6	1.17	Potato	1	155	2	0.1	0.09	[110]
Diisobutyl phthalate	278.34	1.17	Potato	1	155	2	0.1	-0.06	[110]
Diisobutyl phthalate	278.34	1.17	Potato	1	155	2	0.1	-0.17	[110]
Diisobutyl phthalate	278.34	1.17	Potato	1	155	2	0.1	-0.03	[110]
Diisobutyl phthalate	278.34	1.17	Potato	1	155	2	0.1	-0.09	[110]
Dimethyl phthalate	194.18	1.17	Potato	1	155	2	0.1	0.34	[110]
Dimethyl phthalate	194.18	1.17	Potato	1	155	2	0.1	0.38	[110]

Compounds	Molecular Weight	SOM content/%	Plants	Cultivate mode	Exposure time (day)	Protein content (%)	Lipid content (%)	Log RCF	References
Dimethyl phthalate	194.18	1.17	Potato	1	155	2	0.1	0.33	[110]
Dimethyl phthalate	194.18	1.17	Potato	1	155	2	0.1	0.23	[110]
Di-n-butyl phthalate	278.3	3.47	Cabbage	0	50	1.3	0.2	-0.24	[54]
Di-n-butyl phthalate	278.3	3.47	Cabbage	0	50	1.3	0.2	-0.23	[54]
Di-n-butyl phthalate	278.3	3.47	Cabbage	0	50	1.3	0.2	-0.72	[54]
Di-n-butyl phthalate	278.3	3.47	Cabbage	0	50	1.3	0.2	-0.76	[54]
Di-n-butyl phthalate	278.3	3.47	Cabbage	0	50	1.3	0.2	-0.78	[54]
Di-n-butyl phthalate	278.3	3.47	Cabbage	0	50	1.3	0.2	-0.73	[54]
Di(2-Ethylhexyl) phthalate	390.6	3.47	Cabbage	0	50	1.3	0.2	-0.15	[54]
Di(2-Ethylhexyl) phthalate	390.6	3.47	Cabbage	0	50	1.3	0.2	-0.38	[54]
Di(2-Ethylhexyl) phthalate	390.6	3.47	Cabbage	0	50	1.3	0.2	-0.73	[54]
Di(2-Ethylhexyl) phthalate	390.6	3.47	Cabbage	0	50	1.3	0.2	-0.69	[54]
Di(2-Ethylhexyl) phthalate	390.6	3.47	Cabbage	0	50	1.3	0.2	-0.58	[54]
Di(2-Ethylhexyl) phthalate	390.6	3.47	Cabbage	0	50	1.3	0.2	-0.60	[54]
Cresyl diphenyl phosphate	340.3	1.47	Clove	0	1	5.97	13	0.31	[111]
Cresyl diphenyl phosphate	340.3	1.47	Clove	0	3	5.97	13	0.90	[111]
Cresyl diphenyl phosphate	340.3	1.47	Clove	0	5	5.97	13	0.71	[111]
Cresyl diphenyl phosphate	340.3	1.47	Clove	0	7	5.97	13	0.35	[111]
Cresyl diphenyl phosphate	340.3	1.47	Clove	0	9	5.97	13	0.23	[111]
Cresyl diphenyl phosphate	340.3	1.47	Clove	0	11	5.97	13	0.87	[111]
Cresyl diphenyl phosphate	340.3	1.47	Clove	0	13	5.97	13	1.28	[111]
Triphenyl phosphate	326.3	1.47	Clove	0	1	5.97	13	0.13	[111]
Triphenyl phosphate	326.3	1.47	Clove	0	3	5.97	13	0.33	[111]
Triphenyl phosphate	326.3	1.47	Clove	0	5	5.97	13	0.61	[111]
Triphenyl phosphate	326.3	1.47	Clove	0	7	5.97	13	-0.20	[111]

Compounds	Molecular Weight	SOM content/%	Plants	Cultivate mode	Exposure time (day)	Protein content (%)	Lipid content (%)	Log RCF	References
Triphenyl phosphate	326.3	1.47	Clove	0	9	5.97	13	-0.12	[111]
Triphenyl phosphate	326.3	1.47	Clove	0	11	5.97	13	0.39	[111]
Triphenyl phosphate	326.3	1.47	Clove	0	13	5.97	13	0.59	[111]
Tricresyl phosphate	368.4	1.47	Clove	0	7	5.97	13	-0.05	[111]
Tricresyl phosphate	368.4	1.47	Clove	0	9	5.97	13	0.01	[111]
Tricresyl phosphate	368.4	1.47	Clove	0	11	5.97	13	1.25	[111]
Tricresyl phosphate	368.4	1.47	Clove	0	13	5.97	13	1.35	[111]
Cresyl diphenyl phosphate	340.3	1.47	Lemon	0	1	1.5	0.3	0.38	[111]
Cresyl diphenyl phosphate	340.3	1.47	Lemon	0	3	1.5	0.3	0.40	[111]
Cresyl diphenyl phosphate	340.3	1.47	Lemon	0	5	1.5	0.3	0.72	[111]
Cresyl diphenyl phosphate	340.3	1.47	Lemon	0	7	1.5	0.3	1.26	[111]
Cresyl diphenyl phosphate	340.3	1.47	Lemon	0	9	1.5	0.3	0.78	[111]
Cresyl diphenyl phosphate	340.3	1.47	Lemon	0	11	1.5	0.3	1.01	[111]
Cresyl diphenyl phosphate	340.3	1.47	Lemon	0	13	1.5	0.3	1.01	[111]
Triphenyl phosphate	326.3	1.47	Lemon	0	1	1.5	0.3	1.54	[111]
Triphenyl phosphate	326.3	1.47	Lemon	0	3	1.5	0.3	1.81	[111]
Triphenyl phosphate	326.3	1.47	Lemon	0	5	1.5	0.3	0.72	[111]
Triphenyl phosphate	326.3	1.47	Lemon	0	7	1.5	0.3	0.88	[111]
Triphenyl phosphate	326.3	1.47	Lemon	0	9	1.5	0.3	0.28	[111]
Triphenyl phosphate	326.3	1.47	Lemon	0	11	1.5	0.3	1.11	[111]
Triphenyl phosphate	326.3	1.47	Lemon	0	13	1.5	0.3	1.02	[111]
Tricresyl phosphate	368.4	1.47	Lemon	0	7	1.5	0.3	0.65	[111]
Tricresyl phosphate	368.4	1.47	Lemon	0	9	1.5	0.3	-0.07	[111]
Tricresyl phosphate	368.4	1.47	Lemon	0	11	1.5	0.3	0.14	[111]
Tricresyl phosphate	368.4	1.47	Lemon	0	13	1.5	0.3	-0.26	[111]

Compounds	Molecular Weight	SOM content/%	Plants	Cultivate mode	Exposure time (day)	Protein content (%)	Lipid content (%)	Log RCF	References
Cresyl diphenyl phosphate	340.3	1.47	Cape	0	1	1.9	0.7	1.04	[111]
Cresyl diphenyl phosphate	340.3	1.47	Cape	0	3	1.9	0.7	0.92	[111]
Cresyl diphenyl phosphate	340.3	1.47	Cape	0	5	1.9	0.7	1.04	[111]
Cresyl diphenyl phosphate	340.3	1.47	Cape	0	7	1.9	0.7	0.90	[111]
Cresyl diphenyl phosphate	340.3	1.47	Cape	0	9	1.9	0.7	1.72	[111]
Cresyl diphenyl phosphate	340.3	1.47	Cape	0	11	1.9	0.7	1.29	[111]
Cresyl diphenyl phosphate	340.3	1.47	Cape	0	13	1.9	0.7	1.53	[111]
Triphenyl phosphate	326.3	1.47	Cape	0	1	1.9	0.7	0.38	[111]
Triphenyl phosphate	326.3	1.47	Cape	0	3	1.9	0.7	0.59	[111]
Triphenyl phosphate	326.3	1.47	Cape	0	5	1.9	0.7	0.75	[111]
Triphenyl phosphate	326.3	1.47	Cape	0	7	1.9	0.7	0.83	[111]
Triphenyl phosphate	326.3	1.47	Cape	0	9	1.9	0.7	0.99	[111]
Triphenyl phosphate	326.3	1.47	Cape	0	11	1.9	0.7	0.52	[111]
Triphenyl phosphate	326.3	1.47	Cape	0	13	1.9	0.7	0.51	[111]
Tricresyl phosphate	368.4	1.47	Cape	0	9	1.9	0.7	1.77	[111]
Tricresyl phosphate	368.4	1.47	Cape	0	11	1.9	0.7	1.02	[111]
Tricresyl phosphate	368.4	1.47	Cape	0	13	1.9	0.7	0.15	[111]

Table S2 The optimal hyperparameter values for three different model

Machine learning model	The optimal hyperparameter values
GBRT	learning rate: 0.1133, max depth: 23, max features: 45, min samples leaf: 7, min samples spilt: 4, n estimators: 57
RF	max depth: 18, max features: 47, min samples leaf: 2, min samples spilt: 6, n estimators: 69
SVR	C: 5.0024, gamma: auto, kernel: rbf

Table S3 Predicted logRCF values from different models

Actual logRCF	GBRT_Predicted logRCF	RF_Predicted logRCF	SVR_Predicted logRCF	Lasso LR Predicted logRCF
-0.17	-0.351721975	-0.330010239	-0.266332433	-0.324505451
-1.2	-0.289880162	-0.101261768	-0.360962712	0.091057861
0.32	0.108389572	-0.011296608	-0.296675525	0.12592239
-0.84	-0.633755447	-0.453114431	-0.216319803	0.008597265
-0.05	0.070959261	0.066607974	0.12492506	0.124118022
-0.79	-0.828939597	-0.830494053	-0.769583745	-0.827859644
0.06	-0.040415885	0.083590917	-0.280344995	-0.375027748
0.03	0.23125008	0.317326652	0.039702414	0.363114236
-0.23	-0.74892435	-0.719227599	-0.679806153	-0.590059134
-0.81	-0.79625528	-0.793199786	-0.760063755	-0.436053871
-1.38	-1.493891987	-1.335013144	-0.186676457	0.587766955
-0.83	-0.846236188	-0.84547546	-0.810147335	-0.809931357
0.52	0.789537665	0.77994784	0.705078254	0.900058411
-0.07	-0.574554561	-0.577821459	-0.818244857	-0.315675395
1.59	0.964160002	0.735032495	0.809306868	-0.003339433
-0.85	-0.742644051	-0.719272154	-0.669983795	-0.490917631
-0.25	-0.160026544	-0.079539543	-0.125792757	-0.175069479
-0.29	0.009086372	0.057880843	0.08974573	0.443816427
-0.21	0.707345359	0.689487138	0.607544337	0.692094301
-0.84	-0.846236188	-0.84547546	-0.810147335	-0.809931357
-0.12	-0.171218403	-0.24224881	-0.46328657	-0.050659887
-1.73	-1.46894102	-1.36387607	-1.576902944	-1.520690031
0.19	0.240171121	0.160169977	0.07108195	-0.054149064
0.61	0.67589409	0.682503451	0.635926156	0.762053185
-0.28	-0.512818614	-0.538332936	-0.490349916	-0.425780236
-0.77	-0.828939597	-0.830494053	-0.769583745	-0.827859644
-0.89	-0.838630157	-0.839449163	-0.800296213	-0.829251799
0.34	0.332869383	0.382153813	0.39737276	0.305748193
-0.8	-0.846236188	-0.84547546	-0.810147335	-0.809931357
0.13	0.284657852	0.388940735	0.491302091	0.349370058
-0.25	-0.307874091	-0.311886358	-0.339891801	-0.480643996
0.48	0.115248488	0.10749239	0.086017864	0.053830085
0.55	0.808543649	0.729337474	0.656340524	0.630760082
0.19	-0.063142461	0.039828145	-0.074324813	0.185709336
-0.74	-0.127307459	-0.153897807	-0.198191284	-0.160680731
-0.59	-0.332137867	-0.32942738	-0.103277042	-0.249917709
-0.56	-0.791397779	-0.752043583	-0.786095869	-0.70042374
0.76	0.951058836	0.828791076	0.754457115	0.47448332

Actual logRCF	GBRT_Predicted logRCF	RF_Predicted logRCF	SVR_Predicted logRCF	Lasso LR Predicted logRCF
-0.09	-0.10657409	-0.046004203	-0.067255172	-0.01937091
-0.7	-0.471897945	-0.366048414	-0.262420837	-0.23348397
-0.09	-0.152815051	0.006871722	-0.127650178	-0.280819043
0.35	0.457698595	0.586998735	0.798135413	0.761341518
-0.12	-0.222251009	-0.246753549	-0.047313049	-0.100421203
0.88	0.863945343	0.83018696	0.813214538	0.654939078
0.64	0.598429334	0.471246377	0.412462182	0.370982159
0.79	0.887407958	0.794601008	0.64667615	0.473086085
0.5	0.956416487	0.852837257	0.764304802	0.479276932
0.27	0.132776616	-0.066006464	-0.264192196	-0.377470728
-0.58	-0.968339348	-0.829817228	-0.548705609	-0.380875614
-0.23	0.107842562	0.066666379	-0.115481703	-0.171584813
-0.16	-0.129540782	-0.056352361	-0.079672884	-0.265406474
-0.33	-0.32688686	-0.441631815	-0.316864668	-0.252381205
-0.6	-0.513489736	-0.538686367	-0.613085354	-0.445028286
0.78	0.600166945	0.426224223	0.162251598	0.111644759
0.9	1.14651973	1.145509578	1.182415675	0.892418175
-0.02	0.285578128	0.034205607	-0.146313022	-0.192817286
0.18	0.280347395	0.419658213	0.625194992	0.431858409
0.51	0.291703588	0.212932405	-0.287814877	-0.368489083
0.29	0.287502317	0.105038998	0.32914417	-0.383584682
-0.47	-0.32441891	-0.222069228	-0.227327433	-0.098762957
0.38	0.200331963	0.181899359	0.130110324	0.058338693
-0.54	-0.76015587	-0.736926014	-0.923933107	-0.514290107
-0.77	-0.615626735	-0.510916006	-0.643398577	-0.614301831
1.28	0.708603509	0.461075655	0.33764729	0.475023881
0.13	0.022642774	-0.044228723	0.028680115	-0.252866957
1.51	1.149402379	1.011953892	0.803293086	0.705061061
-0.16	-0.143422575	-0.09372351	-0.259721377	-0.379805966
1.48	1.509279133	1.412373309	1.346791423	0.966629932
0.28	1.004677832	1.057076236	0.98342593	0.771451711
-0.77	-0.821654113	-0.821573249	-0.789862022	-0.675068248
0.33	0.200331963	0.181899359	0.130110324	0.058338693
-0.8	-0.980197167	-0.863907284	-0.601648614	-0.385808088
0.18	0.173786515	0.233720696	0.207074619	0.184996889
-0.19	-0.110832283	-0.012422986	-0.078471177	-0.120675916
-0.05	-0.133839147	-0.065595518	-0.087671309	-0.267741712
0.65	1.13643511	0.985371167	0.58858637	0.73263563
-1.56	-1.4580621	-1.402191888	-0.354044344	0.4823075
0.51	-0.169104171	-0.227845063	-0.054346765	0.219596716
-0.77	-0.846236188	-0.84547546	-0.810147335	-0.809931357

Actual logRCF	GBRT_Predicted logRCF	RF_Predicted logRCF	SVR_Predicted logRCF	Lasso LR Predicted logRCF
-0.83	-0.846236188	-0.84547546	-0.810147335	-0.809931357
-0.48	-0.52503234	-0.402861775	-0.450497466	-0.254916499
-0.19	-0.378799193	-0.360537853	-0.339368136	-0.377117592
1.22	1.303775384	1.28561687	1.343614732	0.918363089
0.03	-0.199966416	-0.143252123	-0.192921703	0.311992109
-0.08	-0.440352096	-0.406601876	-0.532558587	-0.085636248
-0.43	-0.689059414	-0.708809071	-0.423201076	-0.400874655
0.29	0.22390118	0.017066989	-0.287713951	-0.357854732
-0.64	-1.02177224	-0.951070934	-0.964031902	-0.825445573
1.04	1.090451351	1.043145649	1.178324426	0.89148408
-0.6	-1.416333025	-1.360789574	-0.767801426	0.475934427
1.53	1.256067949	1.189695914	1.194166582	0.89522046
-0.11	-0.180925847	-0.187261005	-0.153071146	-0.238286616
1.25	1.056264629	0.60794686	1.250812967	0.200163426
0.09	0.024910142	-0.309138979	-0.298522815	-0.097341551
0.65	0.755604701	0.852510399	0.919702252	0.705424446
-0.47	-0.647177846	-0.510290963	-0.827202925	-0.359776621
-0.8	-0.811940347	-0.810261361	-0.749981513	-0.829043932
0.14	-0.349571659	-0.498378009	-0.633687462	-0.712731965
1.36	0.028255626	0.074056791	0.210253628	0.209364663
-0.59	-0.811282271	-0.738888954	-0.906935614	-0.504949156
0.54	0.539952936	0.599774834	0.703646987	0.794649744
0.39	0.154058425	-0.041373269	-0.254925888	-0.382141204
0.28	0.247803949	0.324606604	0.253144786	0.302912874
-0.14	-0.867810748	-0.86155207	-0.8188497	-0.630667809
0.75	0.63481601	0.677296411	0.576696044	0.733770877
-0.84	-0.445572268	-0.294484002	-0.081775868	-0.278105915
-0.7	-0.503433353	-0.349868054	-0.229272132	-0.163174305
-0.84	-0.846236188	-0.84547546	-0.810147335	-0.809931357
0.05	0.373265675	0.577745992	0.677986552	0.437572511
0.18	0.325185665	0.101865214	0.0903925	0.073601361
-0.13	-0.263038699	-0.336698086	-0.274645314	-0.327437168
-0.52	-0.467736125	-0.529413159	-0.422186551	-0.45700995
-0.85	-0.662471435	-0.488504209	-0.432060903	-0.248844881
-1.2	-1.307578526	-1.128617285	-1.152405956	-0.762148592
-0.63	-0.514547044	-0.440305085	-0.235848333	-0.329640869
-0.32	0.186968032	0.093882523	0.131182952	-0.163947058
-0.26	-0.216033506	-0.182646345	-0.190622371	-0.327125565
0.14	-0.003596634	0.017758109	-0.060389267	-0.156172244
-0.79	-0.706773026	-0.669318331	-0.391598956	-0.598910246
0.18	0.646605614	0.3266183	0.177839869	0.485520847

Actual logRCF	GBRT_Predicted logRCF	RF_Predicted logRCF	SVR_Predicted logRCF	Lasso LR Predicted logRCF
0.07	0.179155603	0.055622876	0.103358724	-0.269885873
-0.43	-0.512818614	-0.538332936	-0.490349916	-0.425780236
-0.19	-0.290977123	-0.316965409	-0.314202148	-0.259812234
-0.69	-0.513489736	-0.538686367	-0.613085354	-0.445028286
-0.33	-0.311142909	-0.206734331	0.088252423	0.097273797
-0.43	-0.232094816	-0.246810201	-0.11511643	-0.322207141
-0.47	-0.4800129	-0.54088216	-0.429450201	-0.446375599
-0.85	-0.828939597	-0.830494053	-0.769583745	-0.827859644
-0.33	-0.025303844	0.081941504	-0.276932445	-0.377362986
0.99	0.783659736	0.77973528	0.701495	0.899124316
0.7	-0.045972164	-0.105885738	-0.223829268	-0.236587902
-0.1	-0.426868123	-0.397371761	-0.365723154	-0.52281764
-0.25	-0.307874091	-0.311886358	-0.339891801	-0.480643996
-0.24	-0.307874091	-0.311886358	-0.339891801	-0.480643996
-0.83	-0.846236188	-0.84547546	-0.810147335	-0.809931357
-0.89	-0.846236188	-0.84547546	-0.810147335	-0.809931357
-0.62	-0.775777518	-0.603031631	-0.259303975	-0.187245549
-0.23	-0.257043988	-0.255222656	0.027533224	0.053172571
0.88	1.129353937	1.009838525	0.837272217	0.79132206
0.71	0.863855175	0.68027469	0.543530639	0.373817477
0.15	0.323311348	-0.017488844	0.500908388	-0.512173629
0.3	0.545505716	0.750268288	0.693599612	0.649455167
-0.61	-0.669457867	-0.516158265	-0.622232082	-0.658403058
0.04	-0.008410608	-0.034230747	0.147679469	-0.128395124
-0.36	-0.015348878	-0.085818095	-0.051259929	-0.444037628
0.16	-0.22994029	-0.072222001	0.219937851	-0.432157626
-0.25	-0.369724741	-0.414033238	-0.41751565	-0.574512446
0.14	-0.753625481	-0.636992755	-0.448088936	-0.364181771
-1.52	-0.331597157	-0.368600863	-0.419890913	-0.284042544
0.89	0.647703463	0.682503451	0.617899181	0.753424685
-0.9	-0.815949571	-0.803568146	-0.720314737	-0.814007054
-0.6	-0.158050935	-0.350020626	-0.183880121	-0.053718632
0.3	0.431001546	0.349624067	0.741015903	0.685724295
-0.87	-0.784210652	-0.779205394	-0.689977855	-0.790062002
0.62	0.25025689	0.4614062	0.543194886	0.664770715
-2	-0.177806642	-0.337281668	-0.28752881	-0.067730058
0.01	0.778696338	0.557177364	1.251315997	0.199229331
-0.38	-0.332834797	-0.252491109	-0.209844142	-0.13805711
-0.77	-0.804632715	-0.599613757	-0.256201636	-0.184910311
-0.88	-0.742644051	-0.719272154	-0.669983795	-0.490917631
0.57	1.125725494	1.011953892	0.786394173	0.69643256

Actual logRCF	GBRT_Predicted logRCF	RF_Predicted logRCF	SVR_Predicted logRCF	Lasso LR Predicted logRCF
-0.93	-0.723989298	-0.730587204	-0.770108001	-0.798109811
-0.15	-0.190199483	-0.081567173	-0.011300435	-0.205362449
-0.82	-0.838630157	-0.839449163	-0.800296213	-0.829251799
0	0.173786515	0.233720696	0.207074619	0.184996889
-0.51	-0.465757126	-0.337790639	-0.310260745	-0.062283871
0.34	0.457698595	0.586998735	0.798135413	0.761341518
-0.28	-0.49407365	-0.37007003	-0.279160469	-0.421781918
-1.22	-1.177957278	-0.858719263	-0.69139608	-0.539029779
-0.13	-0.307874091	-0.311886358	-0.339891801	-0.480643996
-0.38	-0.513489736	-0.538686367	-0.613085354	-0.445028286
-0.79	-0.963118347	-0.907628537	-0.415528689	-0.409748558
-0.54	-0.418738396	-0.367158387	-0.272825028	0.093524732
-0.46	-0.73048915	-0.510289264	-0.217114789	-0.150096974
-1.03	-1.007826504	-0.931185922	-0.568890505	-0.683072407
0.71	0.908181454	0.709980234	0.609584472	0.404975953

Text S1 Selected molecular descriptors

1. MATS Descriptors: MATSv5, MATSe5, MATSe6, MATSe8, MATSm5, MATSm6, MATSp4, MATS5m, MATS3e, MATS7e, MATS7i, MATS8i
2. GATS Descriptors: GATSe8, GATSv8, GATS3m, GATS4m, GATS5m, GATS7m, GATS7e, GATS8e, GATS5p, GATS8p, GATS2i, GATS8i, GATS1s, GATS7s
3. VSA and PEOE Descriptors: VSAEstate10, MRVSA9, MRVSA8, PEOEVSA10, PEOEVSA3, PEOEVSA7, PEOEVSA9, EstateVSA4, EstateVSA1
4. Topological and Connectivity Descriptors: dchi0, IC3, chiChain.7, autoCorrelationCharge.4, chiCluster.1, fmf, chiChain.3, carbonTypes.6, longestAliphaticChain, carbonTypes.7
5. Fragment-Based Descriptors: fr_C_O, fr_allylic_oxid, fr_aryl_methyl, fr_bicyclic, fr_unbrch_alkane
6. Miscellaneous Descriptors: nP.1, AATS6i, AATS1s, SpMax5_Bhp, C1SP3, C3SP3, SCH-6, VCH-5, SC-5, VR3_Dt, maxsBr, ETA_Shape_X, ETA_BetaP_s, CIC5.1, nAtomLAC, MDEC-33, LipinskiFailures, topoRadius, GGI10, JGI2, JGI5, VE3_D, VR2_D
7. ATSC and AATSC Descriptors: ATSC3c, ATSC6c, ATSC8c, ATSC6v, ATSC8e, ATSC2p, ATSC3p, ATSC5p, ATSC6i, ATSC3s, ATSC4s, ATSC5s, AATSC4m, AATSC0v, AATSC2v, AATSC3v, AATSC0p, AATSC2i, AATSC3i, AATSC4i, AATSC7s, AATSC8s
8. BCUT Descriptors: bcute10, bcutp5

Text S2 The code of machine learning

(1) t-SNE analysis

```
# Import necessary libraries
import pandas as pd
from sklearn.preprocessing import StandardScaler
from sklearn.manifold import TSNE
import matplotlib.pyplot as plt
from mpl_toolkits.mplot3d import Axes3D
from matplotlib import rcParams

data = pd.read_excel(' data.xlsx')

features = data.drop(columns=['logRCF'])
labels = data['logRCF']

scaler = StandardScaler()
features_scaled = scaler.fit_transform(features)

tsne = TSNE(n_components=3, perplexity=20, learning_rate=200, random_state=42)
tsne_results = tsne.fit_transform(features_scaled)
data['t-SNE-1'] = tsne_results[:, 0]
data['t-SNE-2'] = tsne_results[:, 1]
data['t-SNE-3'] = tsne_results[:, 2]
config = {
    "font.family": 'serif',
    "font.size": 12,
    "mathtext.fontset": 'stix',
    "font.serif": ['Times New Roman'],
    'axes.unicode_minus': False
}
rcParams.update(config)

cm = plt.cm.get_cmap('RdYlGn')
fig = plt.figure(figsize=(12, 10))
ax = fig.add_subplot(111, projection='3d')    # Set the 3D plot projection
sc = ax.scatter(tsne_results[:, 0], tsne_results[:, 1], tsne_results[:, 2], c=labels, cmap=cm, s=50, alpha=0.75)
plt.colorbar(sc, label='logRCF')
ax.set_xlabel('t-SNE 1', fontsize=12)
ax.set_ylabel('t-SNE 2', fontsize=12)
ax.set_zlabel('t-SNE 3', fontsize=12)
plt.title('3D t-SNE')
plt.grid(True)
```

```
plt.show()

data.to_excel('tsne_results.xlsx', index=False)
```

(2) Model construction and interpretation

```
import pandas as pd
import numpy as np
from sklearn.preprocessing import StandardScaler
from sklearn.model_selection import train_test_split, cross_val_score
from hyperopt import fmin, tpe, hp
from sklearn.ensemble import GradientBoostingRegressor
from sklearn.metrics import r2_score, mean_squared_error, mean_absolute_error
import matplotlib.pyplot as plt
import shap
from sklearn.inspection import partial_dependence
from mpl_toolkits.mplot3d import Axes3D
from matplotlib.gridspec import GridSpec
from matplotlib import rcParams

random_seed = 42

data = pd.read_excel("database.xlsx")
X = data.drop(columns=['logRCF'])
y = data['logRCF'].to_numpy()

X_train, X_test, y_train, y_test = train_test_split(X, y, test_size=0.2, 
random_state=random_seed)

scaler = StandardScaler()
X_train = scaler.fit_transform(X_train)
X_test = scaler.transform(X_test)

best_scores = {
    'gradient_boosting': {'score': float('inf'), 'params': None},
}
def objective(params):
    param = params.pop('model')
    model_type = param.pop('type')

    if model_type == 'gradient_boosting':
        model = GradientBoostingRegressor(**param, 
random_state=random_seed)
        score = cross_val_score(model, X_train, y_train, cv=5, scoring='r2').mean()
```

```
        r2 = -score

        if r2 < best_scores[model_type]['score']:
                best_scores[model_type]['score'] = r2
                best_scores[model_type]['params'] = param

        return r2

search_space = {
        'model': hp.choice('model_type', [
                {
                        'type': 'gradient_boosting',
                        'n_estimators': hp.choice('n_estimators_gb', range(30,80)),
                        'learning_rate': hp.uniform('learning_rate_gb', 0.1, 0.2),
                        'max_depth': hp.choice('max_depth_gb', range(15, 25)),
                        'min_samples_split': hp.choice('min_samples_split_gb', range(2, 20)),
                        'min_samples_leaf': hp.choice('min_samples_leaf_gb', range(2, 20)),
                        'max_features': hp.choice('max_features_gb', range(35, 50))
                },
        ])
}

best_params = fmin(fn=objective, space=search_space, algo=tpe.suggest,
max_evals=500, rstate=np.random.default_rng(random_seed))

for model_type, model_info in best_scores.items():
        print(f"Model: {model_type}, Best Score: {model_info['score']}, Best Params:
{model_info['params']}")

print()
for model_type, model_info in best_scores.items():
        model_params = model_info['params'].copy()
        if model_type == 'gradient_boosting':
                model = GradientBoostingRegressor(**model_params,
random_state=random_seed)

        model.fit(X_train, y_train)
        y_pred = model.predict(X_test)

        r2 = r2_score(y_test, y_pred)
        mse = mean_squared_error(y_test, y_pred)
        mae = mean_absolute_error(y_test, y_pred)

        print(f"Model: {model_type:<20}\t R2: {r2:.6f}\t MSE: {mse:.6f}\t MAE:
```

```
{mae:.6f}")
```

# PFI analysis

```
    feature_importance = model.feature_importances_
    feature_importance_df = pd.DataFrame({'Feature': X.columns, 'Importance': feature_importance})
    feature_importance_df = feature_importance_df.sort_values(by='Importance', ascending=False)

    top_15_features = feature_importance_df.head(15)
config = {
    "font.family": 'Arial',
    "font.size": 42,
    "mathtext.fontset": 'stix',
    "font.serif": ['Arial'],
    'axes.unicode_minus': False,
    'axes.labelweight': 'bold',


}
rcParams.update(config)

plt.figure(figsize=(20, 10))
plt.barh(top_15_features['Feature'], top_15_features['Importance'], color='#B54764')
plt.xlabel('Permutaion Importance')
plt.ylabel('Features')
plt.title('The top 15 most important features')
plt.xticks(fontsize=40, fontweight='bold')
plt.yticks(fontsize=40, fontweight='bold')
plt.gca().invert_yaxis()
plt.savefig(f'PFI.png')
```

# SHAP analysis

```
best_model = GradientBoostingRegressor(**best_scores['gradient_boosting']['params'], random_state=random_seed)
best_model.fit(X_train, y_train)

explainer = shap.Explainer(best_model, X_train)
shap_values = explainer.shap_values(X_test, check_additivity=False)

config = {
    "font.family": 'Arial',
    "font.size": 30,
```

```
    "mathtext.fontset": 'stix',
    "font.serif": ['Arial'],
    'axes.unicode_minus': False,
}
rcParams.update(config)

shap.summary_plot(shap_values, X_test, plot_type="dot", feature_names=X.columns,
show=False)
fig = plt.gcf()
fig.set_size_inches(12,8)
plt.xlabel('SHAP Value')
plt.ylabel('Feature')
plt.xticks(fontsize=22, fontweight='bold')
plt.yticks(fontsize=22, fontweight='bold')
plt.savefig(f'SHAP.png')

# ICE analysis
SOM_step_lst = np.arange(SOM_min, SOM_max, 0.01)

SOM_df = pd.DataFrame(index=np.arange(X_test.shape[0]),
columns=list(SOM_step_lst))

for jj in range(X_test.shape[0]):
    for ii, som_val in enumerate(SOM_step_lst):
        SOM_sample = X_test[jj, :].copy()
        SOM_sample[X.columns.get_loc('SOM')] = (som_val -
scaler.mean_[X.columns.get_loc('SOM')]) / scaler.scale_[X.columns.get_loc('SOM')]
        SOM_pred = best_model.predict(SOM_sample.reshape(1, -1))[0]
        SOM_df.iloc[jj, ii] = SOM_pred

config = {
    "font.family": 'Arial',
    "font.size": 16,   #
    "mathtext.fontset": 'stix',
    "font.serif": ['Arial'],
    'axes.unicode_minus': False,
}
rcParams.update(config)

plt.figure(figsize=(10, 6))
for ii in range(SOM_df.shape[0]):
    plt.plot(SOM_step_lst, SOM_df.iloc[ii, :], alpha=0.12, c='#ED630A',
linewidth=0.4)
```

```
SOM_df_mean = SOM_df.mean(axis=0)
plt.plot(SOM_step_lst, SOM_df_mean, alpha=1, c='#ED630A', label="average")

plt.legend(loc=1, prop={'family': 'Arial'})
plt.xlabel('SOM (%)', fontsize=26, family='Arial')
plt.ylabel('Partial Dependence', fontsize=26, family='Arial')
plt.xticks(fontsize=22, fontweight='bold')
plt.yticks(fontsize=22, fontweight='bold')
plt.savefig(f'SOM-ICE.png')
```
